# In-depth quantitative proteomics analysis revealed C1GALT1 depletion in ECC-1 cells mimics an aggressive endometrial cancer phenotype observed in cancer patients with low C1GALT1 expression

**DOI:** 10.1007/s13402-023-00778-w

**Published:** 2023-02-06

**Authors:** Ana Montero-Calle, Álvaro López-Janeiro, Marta L. Mendes, Daniel Perez-Hernandez, Irene Echevarría, Ignacio Ruz-Caracuel, Victoria Heredia-Soto, Marta Mendiola, David Hardisson, Pablo Argüeso, Alberto Peláez-García, Ana Guzman-Aranguez, Rodrigo Barderas

**Affiliations:** 1 Chronic Disease Programme, UFIEC, Instituto de Salud Carlos III, 28220 Majadahonda, Madrid Spain; 2 Department of Pathology, Hospital Universitario La Paz, 28046 Madrid, Spain; 3 Department of Infection and Immunity, Luxembourg Institute of Health, 1445 Strassen, Luxembourg; 4 Biochemistry and Molecular Biology Department, Facultad de Óptica y Optometría, Universidad Complutense de Madrid, 28037 Madrid, Spain; 5 Translational Oncology, La Paz University Hospital (IdiPAZ), 28046 Madrid, Spain; 6 Center for Biomedical Research in the Cancer Network (Centro de Investigación Biomédica en Red de Cáncer, CIBERONC), Instituto de Salud Carlos III, 28046 Madrid, Spain; 7 Molecular Pathology and Therapeutic Targets Group, La Paz University Hospital (IdiPAZ), 28046 Madrid, Spain; 8 Faculty of Medicine, Universidad Autónoma de Madrid, 28029 Madrid, Spain; 9 Tufts Medical Center, Tufts University School of Medicine, Boston, Massachusetts USA; 10 Functional Proteomics Unit, UFIEC, Instituto de Salud Carlos III, 28220 Majadahonda, Madrid Spain

**Keywords:** Endometrial cancer, O-glycosylation, C1GALT1, Quantitative proteomics, SILAC

## Abstract

**Background:**

Endometrial cancer (EC) is the most common cancer of the female reproductive organs. Despite the good overall prognosis of most low-grade ECs, FIGO I and FIGO II patients might experience tumor recurrence and worse prognosis. The study of alterations related to EC pathogenesis might help to get insights into underlying mechanisms involved in EC development and progression.

**Methods:**

Core tumoral samples were used to investigate the role of C1GALT1 in EC by immunohistochemistry (IHC). ECC-1 cells were used as endometrioid EC model to investigate the effect of C1GALT1 depletion using C1GALT1 specific shRNAs. SILAC quantitative proteomics analyses and cell-based assays, PCR, qPCR, WB, dot-blot and IHC analyses were used to identify, quantify and validate dysregulation of proteins.

**Results:**

Low C1GALT1 protein expression levels associate to a more aggressive phenotype of EC. Out of 5208 proteins identified and quantified by LC-MS/MS, 100 proteins showed dysregulation (log_2_fold-change ≥ 0.58 or ≤-0.58) in the cell protein extracts and 144 in the secretome of C1GALT1 depleted ECC-1 cells. Nine dysregulated proteins were validated. Bioinformatics analyses pointed out to an increase in pathways associated with an aggressive phenotype. This finding was corroborated by loss-of-function cell-based assays demonstrating higher proliferation, invasion, migration, colony formation and angiogenesis capacity in C1GALT1 depleted cells. These effects were associated to the overexpression of ANXA1, as demonstrated by ANXA1 transient silencing cell-based assays, and thus, correlating C1GALT and ANXA1 protein expression and biological effects. Finally, the negative protein expression correlation found by proteomics between C1GALT1 and LGALS3 was confirmed by IHC.

**Conclusion:**

C1GALT1 stably depleted ECC-1 cells mimic an EC aggressive phenotype observed in patients and might be useful for the identification and validation of EC markers of progression.

**Supplementary Information:**

The online version contains supplementary material available at 10.1007/s13402-023-00778-w.

## Introduction

Endometrial cancer (EC) is the most common cancer of female reproductive organs, in which cancerous cells lining the uterus might invade the neighboring myometrium, or metastasize to lymph nodes, or distal organs, mainly vagina, ovaries, and lungs [1]. Endometrial carcinomas can be categorized according to their histologic type and grade of differentiation [2]. Endometrioid endometrial cancer is the most common histologic type and can show various grades of differentiation. Well-differentiated (low-grade or G1/G2) endometrioid tumors demonstrate less than 50% of solid non-squamous areas and represent the most commonly diagnosed endometrial tumor. In addition, these low-grade endometrioid tumors are associated with low risk of relapse and metastasis [3, 4]. However, their high grade endometrioid counterparts (G3) show more than 50% of solid non-squamous areas, and are associated with high risk of relapse and metastasis [5]. Aside from endometrioid tumors, other high grade (G3) histologic types encountered include serous, clear-cell and undifferentiated carcinomas [4, 6]. According to the extent of the disease, ECs are classified following the FIGO (International Federation of Gynecology and Obstetrics) staging system [1, 6, 7]. Tumors confined to the uterus and cervix are categorized as FIGO stage I or II, while those tumors extending to lymph nodes or distant organs are considered FIGO stage III and IV categories [6]. In addition, despite the good overall prognosis of most EC patients, some FIGO I and FIGO II patients might also experience tumor recurrence after surgery [8].

Glycosylation is one of the most common post-translational modifications of proteins [9]. Since glycans are involved in different tumorigenic pathways, such as cell signaling, angiogenesis, invasion, metastasis or immune modulation, previous works have reported an important role of aberrant glycosylation in cancer development [9–11]. O-linked glycosylation is mainly directed to the hydroxyl oxygen of serine (S) and threonine (T) residues [10], and is of great interest for the study of cancer progression and development as many tumor-associated antigens are derived from this process [9, 12]. In endometrial cancer, alterations in the expression of GalNAc-T6 have been associated with prognosis [13, 14]. In addition, increased levels of O-GlcNAc glycosyltransferase enzymes have been associated with the grade of differentiation of these tumors [15, 16]. C1GALT1 (T-synthase or Core 1β3 Galactosyl Transferase) is a primary enzyme in the biosynthesis of core 1 O-glycans and catalyzes the transference of galactose to the Tn Antigen (GalNAcα1-Ser/Thr) [9, 12, 17], resulting in the formation of the T Antigen (Galß1–3GalNAcα1-Ser/Thr) [17]. Both Tn and T antigen O-glycans have been described as oncofetal antigens involved in the development of different cancers [12, 18]. In addition, C1GALT1 has also been associated with prognosis in other cancers [9, 19].

In this study, we have shown by immunohistochemistry (IHC) that the more aggressive ECs showed low protein expression levels of C1GALT1. These results were confirmed at transcriptomic and proteomic level using the CPTAC data. Thus, we aimed to analyze the effect of C1GALT1 depletion in EC by quantitative proteomics. To this end, ECC-1 cells were used as a model of endometrioid EC, and the effect of C1GALT1 depletion on these cells using short hairpin RNAs (shRNA) was analyzed by SILAC quantitative proteomics analyses. *Forward* and *Reverse* SILAC experiments were performed for the identification of changes in the cell extract and secretome proteomes associated to the depletion of C1GALT1 in EC. In total, 5208 and 3616 proteins were identified and quantified in the cell extract and secretome proteomes by LC-MS/MS, respectively. Of them, 100 and 144 proteins showed dysregulation with a log_2_fold-change ≥ 0.58 or ≤-0.58 in C1GALT1 stably depleted ECC-1 cells’ extracts and secretomes, respectively. Dysregulation of selected proteins was validated by PCR, real-time quantitative PCR (qPCR), western blot (WB), dot blot and immunofluorescence (IF) using cell extracts, secretome and exosomes from the ECC-1 cell lines. Moreover, loss-of-function cell-based assays confirmed the observed association of low C1GALT1 expression with an aggressive phenotype in EC by bioinformatics, demonstrating the usefulness of the cell model mimicking an aggressive phenotype of EC. In addition, ANXA1 silencing reversed partially the aggressive phenotype of shC1GALT1 ECC-1 cells. Finally, the association of LGALS3 (Galectin-3) dysregulation to C1GALT1 expression levels and EC grade was confirmed by IHC.

## Materials and methods

### Tissue microarrays (TMA) and immunohistochemistry (IHC)

79 patients diagnosed and surgically treated at University Hospital La Paz between 2003 and 2015 were included in the present study. Formalin fixed paraffin embedded (FFPE) tissue blocks from radical hysterectomy specimens were reviewed by an expert pathologist. From each tissue block 1 to 4 cores from viable tissue areas were obtained to build tissue microarrays (TMA) [20]. Immunohistochemistry (IHC) was performed on 4 μm tissue slices using specific antibodies of C1GALT1 (1:100, SCBT #sc-100,745) and LGALS3 (1:100, Gennova #GAL3-388) on an OMNIS autostainer platform (Dako). Afterwards, tissue cores were scored by an expert pathologist. Cores were considered adequate for analysis if at least 25% of tumoral tissue area per spot was available for scoring. The percentage of cytoplasmic C1GALT1 or LGALS3 positive tumor cells was assessed in an 11-tiered scale (ranging from 0 to 100% in 10% increments). In addition, the average C1GALT or LGALS3 staining intensity was recorded in a 4-tiered scale (absent/faint/moderate/intense). Finally, C1GALT or LGALS3 expression score was obtained by multiplying the percentage of expressing tumor cells by the average tumor staining intensity.

### CPTAC data, ssGSEA and bioinformatics analysis

Clinical, proteomic TMT-plex and transcriptomic data from the UCEC-CPTAC study were accessed through LinkedOmics data portal [21, 22]. Samples with low tumor purity or belonging to patients who had received neoadjuvant treatment were removed from downstream analyses. In addition, carcinosarcomas were also discarded before proceeding with the analysis.

Single-sample Gene Set Enrichment Analysis (ssGSEA) was then performed using the GSVA R package (version 4.1) [23]. Gene Ontology, KEGG pathways and Human Reactome gene set collections were accessed through the msigdbr R package (version 7.4.1) [24]. Gene sets were screened, retaining pathways associated with glycosylation, endometrial cancer and vascular remodeling. Further, gene sets were filtered, and pathways that were weakly represented in the proteome data (< 4 members and < 20% of pathway members quantified) were removed from the analysis. After pathway scoring, correlation between pathway activity and C1GALT1 protein expression was assessed using Pearson correlation coefficients.

STRING (version 11.0), Reactome Pathway Database and DAVID (version 6.8) were used to study protein enrichment and to identify the altered networks and pathways in which proteins identified as dysregulated by C1GALT1 depletion are involved [25–27]. STRING settings were fixed to MCL clustering enrichment 2 and 0.4 confidence score. Venn diagram was obtained with R (version 3.6.2), using “Venn” package.

### Cells and C1GALT1 stably depletion

The epithelial ECC-1 cell line from the American Type Culture Collection (ATCC; Manassas, VA) derived from an endometrioid adenocarcinoma was used in this study [28, 29].

ECC-1 cells were stably depleted for C1GALT1 and Scrambled using retroviruses constructed using the pSUPER.retro.puro vector system, as previously described [30]. Three double-stranded hairpin oligonucleotides designed to target the human C1GALT1 gene (shRNA target sequences (SEQ) 1 (CAAACACGTCAAAGCTACT), 2 (TAAGCAAAGAAGCCTTGAA), and 3 (TACAGATATCAACCTACCT), cloned into pSUPER.retro.puro vector were used. A scrambled sequence (GCGCGCTTTGTAGGATTCG) was used as a control.

### Stable isotope labeling by amino acids in cell culture (SILAC)

SCRAMBLE and shC1GALT1 ECC-1 cells were grown in 10% FBS DMEM at 37ºC and 5% CO_2_ until 90% confluence. ECC-1 cells were harvested with trypsin-EDTA (Lonza), and two 25 cm^2^ cell culture flasks (Corning, USA) per cell line were seeded with 25,000 cells and incubated 24 h at 37ºC and 5% CO_2_ in 10% FBS DMEM (Dulbecco’s modified Eagle’s medium). Then, SCRAMBLE and shC1GALT1 ECC-1 cells were separately incubated with 5 mL of DMEM [^12^C_6_]-L-arginine and [^12^C_6_]-L-lysine (R0K0, light medium, GeminiBio) or DMEM [^13^C_6_]-L-arginine and [^4,4,5,5^D_4_]-L-lysine (R6K4, heavy medium, GeminiBio) supplemented with 10% dialyzed FBS (GeminiBio). Both cell lines were labeled with heavy and light mediums to perform label swapping SILAC forward and reverse experiments as biological and technical replicates, as previously described [31]. After 8 cell doublings to ensure > 98% incorporation of labeled amino acids, 2 × 10^6^ labelled cells were seeded in 25 cm^2^ cell culture flasks (Corning, USA) and incubated at 37ºC and 5% CO_2_ in R0K0 or R6K4 supplemented with 10% dialyzed FBS until 90% confluence. Then, cells were washed 3 times with PBS 1x and incubated 1 h with R0K0 or R6K4 medium without FBS to remove all traces of FBS. Next, cells were washed 3 times with PBS 1x and incubated 48 h 37ºC and 5% CO_2_ with 5 mL of R0K0 or R6K4 DMEM-free serum. Then, conditioned media (secretomes) were collected and centrifuged 5 min at 1200 rpm and RT to remove cell debris. In addition, cells were harvested with 4 mM EDTA-PBS, centrifuged 5 min at 1200 rpm and lysed with RIPA buffer to analyze the total cell extracts.

### Trypsin digestion and fractionation

Secretomes and cell extracts were separately analyzed. For each proteome analysis, two SILAC *forward* and *reverse* experiments were analyzed. The *forward* experiment analyzed R6K4 shC1GALT1 ECC-1 cells and R0K0 SCRAMBLE ECC-1 cells, whereas the *reverse* experiment compared R0K0 shC1GALT1 ECC-1 cells and R6K4 SCRAMBLE ECC-1 R6K4, as biological and technical replicates.

For SILAC experiments, 40 µg of each protein extract were pooled together, methanol-chloroform precipitated and the 80 µg of proteins resuspended in 100 µL of 0.1 M TEAB. Then, proteins were reduced with 10 µL 100 mM tris(2-carboxyethyl)phosphine (TCEP, Sigma Aldrich) during 45 min at 37ºC and 600 rpm and alkylated with 11 µL of 0.4 M chloroacetamide (Sigma Aldrich) during 30 min at room temperature (RT) in darkness. Next, proteins were digested in-solution with 3.2 µg of porcine trypsin (Thermo Fisher Scientific) in 20 mM HEPES pH 8.0 at 37ºC and 600 rpm during 14 h. The day after, samples were dried under vacuum prior to separation using High pH Reversed-Phase Peptide Fractionation Kit (Pierce). Desiccated samples were reconstituted in 300 µl H_2_O Milli-Q, TFA 0.1%, applied to the columns and peptides fractionated according to the manufacturer’s instructions. In total, twelve fractions were obtained for each SILAC experiment, directly dried under vacuum and stored at -80ºC until analysis in LC-MS/MS runs using a Q Exactive HF-X hybrid quadrupole-Orbitrap mass spectrometer (Thermo Fisher Scientific).

### LC-MS/MS analysis

Peptide separations were carried out on an Easy-nLC 1200 nano system (Thermo Fisher Scientific). For each analysis, samples were loaded into a precolumn Acclaim PepMap 100 (Thermo Fisher Scientific) and eluted in a RSLC PepMap C18, 15 cm long, 50 μm inner diameter and 1.9 μm particle size (Thermo Fisher Scientific). The mobile phase flow rate was 300 nl/min using 0.1% formic acid in water (solvent A) and 0.1% formic acid in acetonitrile (solvent B). The gradient profile was set as follows: 0-35% solvent B for 124 min, 35-90% solvent B for 4 min, 20% solvent B for 17 min. Four microliters of each sample were injected.

For ionization, 1800 V of liquid junction voltage and 275 °C capillary temperature was used. The full scan method employed a m/z 350–1700 mass selection, an Orbitrap resolution of 70,000 (at m/z 200), a target automatic gain control (AGC) value of 3e6, and maximum injection times of 100 ms. After the survey scan, the 12 most intense precursor ions were selected for MS/MS fragmentation. Fragmentation was performed with a normalized collision energy of 27 and MS/MS scans were acquired with a starting mass of m/z 100, AGC target was 2e5, resolution of 17,500 (at m/z 200), intensity threshold of 8e3, isolation window of 2 m/z units and maximum IT was 100 ms. Charge state screening was enabled to reject unassigned, singly charged, and greater than or equal to seven protonated ions. A dynamic exclusion time of 20 s was used to discriminate against previously selected ions.

### MS data analysis

MS data were analyzed with MaxQuant (version 1.6.6.0) using standardized workflows. Mass spectra *.raw files were searched against Uniprot UP000005640_9606.fasta Homo sapiens (human) 2019 database (20,962 protein entries, downloaded: May 2019) using standard type. Trypsin/P was specified as cleavage enzyme, allowing a mass tolerance of 20 ppm (Orbitrap). Precursor and reporter mass tolerance were set to 4.5 ppm and 0.003 Da, respectively, allowing 2 missed tryptic cleavages. Carbamidomethylation of cysteines was set as a fixed modification, and methionine oxidation, N-terminal acetylation and Ser, Thr and Tyr phosphorylation were set as variable modifications. Unique and Razor peptides were considered for quantification. Minimal peptide length and maximal peptide mass were fixed to 7 amino acids and 4600 Da, respectively. Identified peptides were filtered by their precursor intensity fraction with a FDR threshold of 0.01. Proteins identified with at least one peptide and an ion score above 99% were considered for evaluation, whereas proteins identified as potential contaminants were excluded from the analysis.

Proteins identified and quantified with one or more peptides and a log_2_fold-change ≥ 0.58 or ≤-0.58 were selected as dysregulated proteins by the depletion of C1GALT1 in ECC-1 cells (upregulated ≥ 0.58, or downregulated ≤-0.58). The fold change cutoff for dysregulated proteins was calculated using a permutation-based test [32], as previously done [33–35].

### Exosome isolation and purification

Extracellular vesicles released by endometrial ECC-1 cells were isolated via the differential centrifugation of the conditioned media (secretome) [36]. In brief, five 175 cm^2^ cell culture flasks (Corning, USA) were seeded per cell line and cells were grown until 90% confluence at 37ºC and under 5% CO_2_. To avoid exogenous exosome contamination, cells were washed with PBS, incubated for 1 h in FBS-free DMEM, washed with PBS, and incubated with 20 mL of DMEM without FBS for 48 h at 37ºC under 5% CO_2_. Then, 100 mL of conditioned medium per cell line were centrifuged at 500 g for 5 min at 4ºC to remove the cell debris (pellet) and then centrifuged again at 2000 g for 10 min at 4ºC to remove vesicles greater than 1 μm (pellet). Next, exosomes were purified by differential centrifugation (Beckman-Coulter ultracentrifuge, XL-100 K, USA) [36]. Exosomes were analyzed by a NanoSight NS300 (Malvern Panalytical, United Kingdom) and by transmission electron microscopy using a FEI Tecnai 12 electron microscope to verify the quality of the purified exosomes, as previously described [36]. Exosomes were stored at -80ºC until use.

### PCR and real-time quantitative PCR (qPCR)

For RNA extraction, cell pellets from SCRAMBLE and shC1GALT1 ECC-1 cells were incubated with 500 µL of NZYol (NZYtech) during 5 min at RT for cell disaggregation, incubated with 100 µL of chloroform (Sigma Aldrich) during 3 min at RT and centrifuged 15 min at 12,000 *g* and 4ºC and the upper phase, containing the RNA, transferred to a new tube. RNA samples were purified using the RNeasy Mini Kit (Qiagen) following manufacturer’s instructions. Purified total RNA was quantified with the Nanodrop 2000 C (Thermo Fisher).

cDNA was obtained from 1 µg of total RNA using the NZY First-Strand cDNA Synthesis Kit (NZYtech) following manufacturer’s instructions, and directly used for the quantification of mRNA levels of selected genes after C1GALT1 depletion. PCR analysis was performed using Phusion High-Fidelity DNA Polymerase (Thermo Scientific) and the corresponding specific oligonucleotides (Supplementary Table 3). qPCRs analyses were performed onto the Light Cycler 480 (Roche) (40 cycles at 65ºC) using the TB Green Premix Ex Taq II (Takara) and the corresponding specific oligonucleotides (Supplementary Table 3). mRNA levels of 18S were used for normalization.

### Protein extraction and quantification

ECC-1 SCRAMBLE and shC1GALT1 cells were lysed in 500 µL of lysis buffer (RIPA, Sigma Aldrich) supplemented with 1x protease and phosphatase inhibitors (MedChemExpress) using 16G and 18G needle syringes until homogeneity. Then, samples were centrifuged at 10,000 g and 4ºC during 10 min and protein extracts (supernatants) collected and stored at -80ºC until use. In addition, secretome proteins were methanol-chloroform precipitated and re-suspended in lysis buffer supplemented with 1x protease and phosphatase inhibitors (MedChemExpress) prior to its use.

Protein concentration was determined by Trp quantification method [37]. Protein concentration of exosomes was quantified using a MicroBCA Protein Assay Kit (Thermo Fisher Scientific, USA) following manufacturer’s instructions. For SDS-PAGE analysis, exosomes were lysed with loading buffer containing 1.5% β-mercaptoethanol (five cycles of 5 min on ice and 5 min at 95ºC).

### Western blot and Dot blot

For western blot (WB) analysis, 10–15 µg of each protein extract were separated on 10% SDS-PAGE under reducing conditions and transferred to nitrocellulose membranes at 100 V during 90 min. 40–60 µg of secretome proteins in 100 µL PBS were dot blotted onto nitrocellulose membranes using the Bio-Dot 96-Well Microfiltration (Bio-Rad).

Then, membranes were blocked with 0.1% Tween PBS 1x containing 3% skimmed milk (blocking buffer) during 1 h at RT and incubated with primary antibodies at optimized dilutions (Supplementary Table 4) in blocking buffer O/N at 4ºC. Then, membranes were washed three times with 0.1% Tween PBS 1x and incubated with the appropriate indicated HRP-conjugated secondary antibodies (Supplementary Table 4) diluted in blocking buffer during 1 h at RT. Next, membranes were washed three times with 0.1% Tween PBS, and, finally, signal was developed using the ECL Pico Plus chemiluminescent reagent (Thermo Fisher Scientific) and detected on an Amersham Imager 680 (GE Healthcare). Protein band intensities were quantified using ImageJ Software.

### Immunofluorescence

For IF, ECC-1 cells were harvested with Trypsin-EDTA (Lonza) and 1 × 10^5^ cells were seeded per crystal slide ON at 37ºC and 5% CO_2_. Then, cells were fixed with 4% paraformaldehyde for 20 min at 37 ºC, washed 3 times with PBS 1x and permeabilized with 0.1% Triton X-100 PBS 1x for 10 min at RT. Cells were then washed 3 times with PBS 1x, blocked with 10% FBS 0.1% Tween-20 PBS 1x for 1 h at RT, and incubated with the corresponding antibody 1:50 diluted in 10% FBS 0.1% Tween-20 PBS 1x O/N at 4ºC (Supplementary Table 4). Then, cells were washed 3 times with PBS 1x and incubated with the appropriate fluorophore conjugated secondary antibodies for 1 h at RT (Supplementary Table 4), followed by incubation with 1 µg/mL Hoechst (Hoechst 33,342 Solution (20 mM) Thermo Scientific) 1:1000 diluted in 10% FBS 0.1% Tween-20 PBS 1x for 15 min at RT. Cells were observed with a confocal microscope (Leica TCS SP5, Leica Microsystems). Images were acquired with a 63x oil immersion N.A. 1.2 objective using the Leica Confocal Software (Las X). All images were acquired in the same conditions (pixel size, z-stack size, excitation laser power and detector sensitivity).

### Cell-based assays

Cell-based assays of stably depleted C1GALT1 ECC-1 cells in comparison to SCRAMBLE ECC-1 cells were performed as previously described [38–40]. In brief, proliferation assays were performed with the MTT reagent (Sigma Aldrich). ECC-1 cells were harvested with Trypsin-EDTA (Lonza) and 5 × 10^3^ cells per well were seeded in 96-well plates (Corning) in 10% FBS DMEM in quadruplicated. The day after, growth medium was removed and 100 µL of 5% FBS DMEM was added to each well, and incubated during 72 h at 37ºC and 5% CO_2_. Then, cells were incubated at 37ºC and 5% CO_2_ for 1 h with 1 mg/mL MTT solution in DMEM. Finally, MTT solution was removed; cells were washed with PBS 1x and lysed with 50 µL of 100% DMSO (Merck). Subsequently absorbance was read at 570 nm using a Spark multimode microplate (TECAN).

For cell adhesive properties analysis, ECC-1 cells were incubated in DMEM free serum during 24 h at 37ºC and 5% CO_2_. 96-well plates (Corning) coated with 100 µL 0.1 M NaHCO_3_ containing Matrigel matrix (Sigma Aldrich, 0.4 µg /mm^2^) were incubated overnight (O/N) at 4ºC. Then, 96-well plates were blocked with 200 µL of sterile DMEM 0.5% BSA (adhesion medium) during 2 h at 37ºC and ECC-1 cells separately stained with 1 mg/mL BCEBF (Sigma) diluted 1:100 in DMEM during 30 min at 37ºC and 5% CO_2_. Next, cells were harvested with 4 mM EDTA-PBS, and 1 × 10^5^ cells in 50 µL of adhesion medium were transferred to each pre-coated well with Matrigel in quadruplicate. Cells were incubated for 2 h at 37ºC and 5% CO_2_, and, then wells were washed twice with 100 µL PBS 1x to remove non-attached cells. Finally, attached cells were lysed with 50 µL of 10% SDS in PBS, and fluorescence signal read at 436-535 nm excitation-emission, respectively, with the Spark multimode microplate (TECAN).

For invasion analyses, 6.5 mm transwells with 8.0 μm Pore Polycarbonate Membrane Inserts (Corning) were placed onto 24-well plates (Corning) and coated with 50 µL of Matrigel matrix diluted in 1:3 in DMEM and incubated at 37ºC for 1 h. ECC-1 cells were harvested with Trypsin-EDTA (Lonza) and 1 × 10^6^ cells were re-suspended in 100 µL sterile adhesion medium and transferred to pre-coated transwells, in duplicate. As chemoattractant, 700 µL of 10% FBS DMEM was used. Then, cells were incubated during 22 h at 37ºC and 5% CO_2_. Non-invading cells and Matrigel were removed from the upper surface of the transwells’ membrane, and invasive cells on the lower membrane were fixed with 1 mL of 4% paraformaldehyde for 1 h at RT. Finally, cells were stained with 1 mL of 0.2% crystal violet 25% methanol during 30 min at RT, washed several times with Milli-Q H_2_O (H_2_Omq) to remove dye traces, photographed with the DMi1 Microscope (Leica) and counted with ImageJ program (Fiji).

The migratory capacity of ECC-1 cells was evaluated using 2-well silicone inserts (Ibidi). Inserts were placed on 24-well plate (Corning). Then, ECC-1 cells were harvested with Trypsin-EDTA (Lonza), 2.5 × 10^5^ cells were resuspended in 70 µL of 10% FBS DMEM, seeded in each well of the inserts and incubated O/N at 37ºC and 5% CO_2_. Each cell line was analyzed in duplicate. The next day, inserts were removed, and 1 mL of 10% FBS DMEM was added to each well and the wound monitored on a Thunder imager (Leica) at 37ºC and 5% CO_2_ by snapshotting the wounds every hour for 48 h. Finally, images were processed with the ImageJ program (Fiji) and the MRI Wound Healing Tool.

For colony soft agar assay, sterilized 5% and 0.5% noble agar in H_2_Omq was used. 2 mL of 5% noble agar solution 1:10 diluted in 10% FBS DMEM were solidified on 6-well plates during 15 min at RT. Subsequently, ECC-1 cells were harvested with Trypsin-EDTA (Lonza), and 25,000 cells in 1 mL of 10% FBS DMEM were 1:2 diluted in 0.5% noble agar solution, and placed onto the solidified agar in duplicate. Cells were incubated at RT for 30 min to allow agar solidification, and then incubated at 37ºC and 5% CO_2_ for 4 weeks for colony formation. 100 µL of 10% FBS DMEM were added to each well once a week. Wells were photographed with the DMi1 Microscope (Leica) and colonies counted with ImageJ program (Fiji).

Human umbilical vein endothelial cells (HUVEC) cells were used for tube formation assay using the secretome of shC1GALT1 and SCRAMBLE ECC-1 cells harvested after 48 h of incubation in Endothelial cell growth medium 2 (EGM-2) supplemented with growth factors (Hydrocortisone, hFGF-B, VEGF, R3-IGF-1, Ascorbic acid, hEGF, GA-100 and Heparin) without FBS. HUVEC cells were grown at 37ºC and 5% CO_2_ until 90% confluence in 10% FBS EGM-2 medium, and incubated 24 h in complete EGM-2 medium without FBS. 96-well plates were coated with 30 µL of Matrigel matrix (Sigma Aldrich) per well and incubated 1 h at 37ºC. Then, HUVEC cells were harvested with trypsin-EDTA (Lonza) and 20,000 or 40,000 HUVEC cells were seeded per well in EGM-2 medium without FBS and 1/2 diluted with the secretome of shC1GALT1 or SCRAMBLE ECC-1 cells. Plates were incubated at 37ºC and 5% CO_2_ during 6 h, and tube formation was monitored each 2 h with the DMi1 Microscope (Leica). Images were processed with the ImageJ program (Fiji) and the angiogenesis analyzer tool.

### Transient ANXA1 Silencing

For transient ANXA1 silencing, transfection was performed in 6-well plates using the jetPRIME reagent (PolyPlus Transfection) with, alternatively, ON-TARGETplus Human ANXA1 (301) siRNA (J-011161-07-0010; Dharmacon) or control siRNAs (SIC001; Sigma-Aldrich) on shC1GALT1 and SCRAMBLE ECC-1 cells, according to established protocols [41, 42]. Briefly, 2.5 × 10^5^ cells were transfected with 22 pmol siRNA using 2 µl of JetPRIME Transfection reagent and 100 µl of JetPRIME buffer (PolyPlus Transfection). Then, 48 h after transfection, cells were analyzed by PCR, qPCR and WB. Alternatively, 24 h after transfection cells were used for indicated cell-based assays as above.

## Results

### C1GALT1 IHC evaluation and CPTAC scoring

**Fig. 1:**
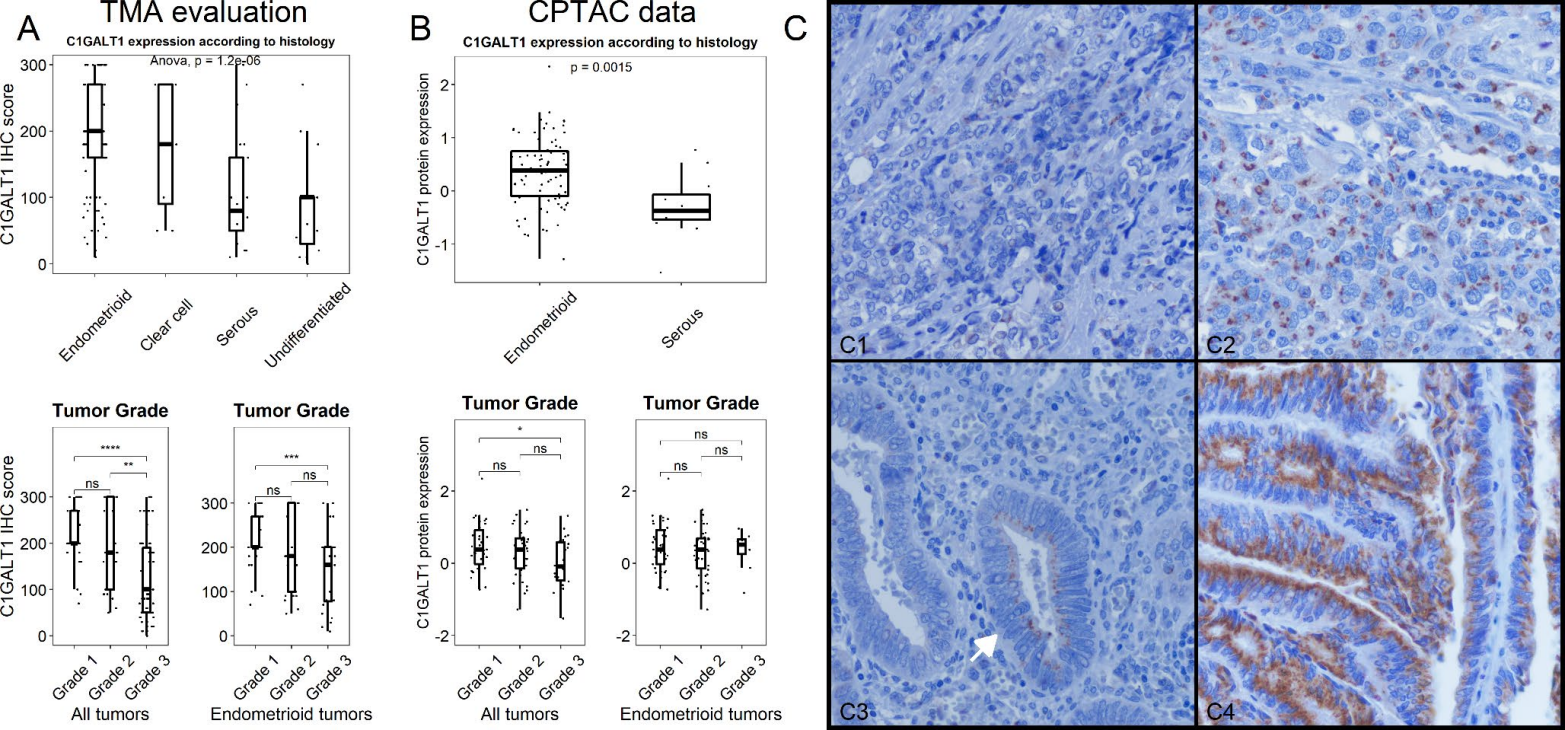
Analysis of C1GALT1 expression in endometrial cancer. **A**, Immunohistochemistry analysis of 159 EC tissue samples - endometrioid (63.5%), serous (18.9%) and clear cells or undifferentiated (17.6%) histology- showed a downregulation of C1GALT1 expression in parallel to the aggressiveness of EC. **B**, CPTAC-UCEC study composed by 97 tumor samples (83 endometrioid, 13 serous and 1 clear cell samples) confirmed the downregulation of C1GALT1 expression in aggressive EC. **C**, Morphologic assessment of C1GALT1 IHC staining revealed according to the protein staining pattern, a complete absence (1.25%), a variable expression without polarity (46.54%) and an apical-side polarized expression (52.2%) of C1GALT1. *, p value < 0.05; **, p value < 0.001; ***, p value < 0.0001; ****, p value < 0.00001

A total of 159 out of 178 cores from 79 EC patients, which represents all major histologic EC types, were considered adequate for cytoplasmic C1GALT1 expression assessment by IHC. Out of the 159 cores, 101 (63.5%) belonged to endometrioid histology, 30 (18.9%) were serous and the remaining 28 (17.6%) belonged to clear cell or undifferentiated histology. Among endometrioid carcinoma cores, 65 (64.4%) belonged to low grade (FIGO grade 1 or 2) carcinomas, while 36 (35.6%) were high grade. C1GALT1 score varied across histologic types, with endometrioid tumors displaying the highest protein expression (median C1GALT1 IHC score = 200). On the other hand, aggressive histologic variants (clear cell, serous and undifferentiated) showed lower C1GALT1 IHC scores (p value < 0.001) (Fig. 1 A). In addition, C1GALT1 expression negatively correlated with tumor grade. This finding was also detected when analyzing endometrioid tumors independently.

Next, data from the CPTAC-UCEC study were analyzed to further verify the C1GALT1 protein expression in an independent cohort of 97 EC samples. Among them, 83 were classified as endometrioid (85.6%), 13 (13.4%) as serous and one as clear cell carcinoma (1%). *In silico* analysis of C1GALT1 protein expression measured by quantitative TMT-proteomics resulted in similar results to our IHC assessment (Fig. 1B). First, serous carcinomas showed significantly lower C1GALT1 protein expression compared with endometrioid carcinomas (median C1GALT1 expression = -0.37 vs. 0.385). Second, a significant trend towards lower C1GALT1 expression in high grade tumors was also evident, in spite of this association being less obvious and non-significant when analyzing endometrioid tumors independently. In addition, transcriptomic data from the CPTAC cohort further supported C1GALT1 expression findings (Fig. S1). Median C1GALT mRNA expression was significantly lower in serous carcinomas compared to endometrioid tumors. Similarly, high-grade tumors showed a non-significant lower C1GALT mRNA expression trend compared to their low-grade counterparts.

Finally, morphologic assessment of C1GALT1 IHC staining characteristics revealed three different staining patterns (Fig. 1 C). First, a small subset of samples (2/159 cores) demonstrated a complete absence of C1GALT1 protein expression. Second, nearly half of samples (74/159 cores) showed variable C1GALT1 protein expression levels without evident protein expression polarity within the cell. Finally, 83 out of 159 cores demonstrated that C1GALT1 protein expression was polarized towards the apical side of the tumor cell. Noticeable, cores showing polarized C1GALT1 expression demonstrated higher C1GALT1 IHC scores compared to non-polarized expressing samples (median 200 vs. 95, p value < 0.001).

Collectively, these results analyzing 176 EC samples − 79 EC patients from the University Hospital La Paz cohort and 97 EC patients from the CPTAC cohort- demonstrated an association of low C1GALT1 protein expression with an aggressive EC phenotype.

### Identification of dysregulated proteins associated with C1GALT1 depletion

We next proceeded to analyze by quantitative proteomics the effect of C1GALT1 depletion on endometrioid ECC-1 cells as a model of aggressive EC phenotype for the elucidation of new mechanisms underlying the disease.

**Fig. 2:**
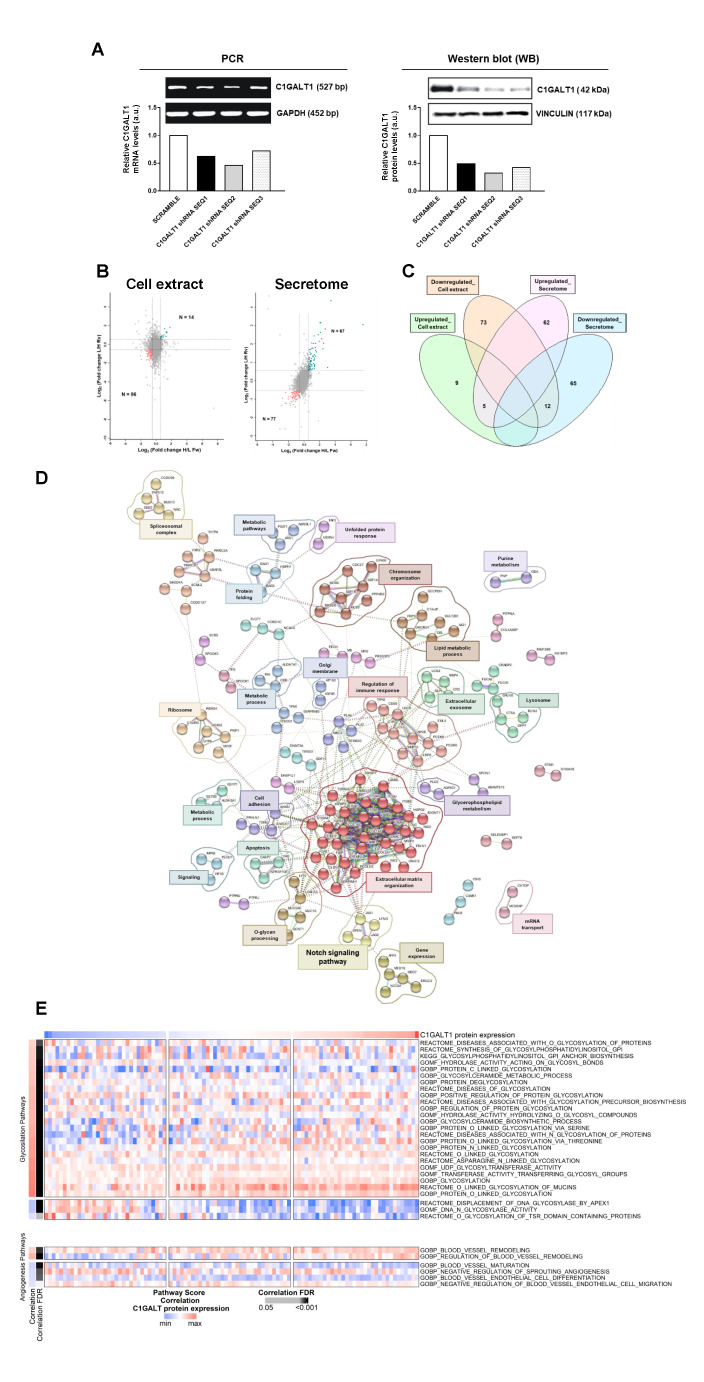
Proteomic and bioinformatics analysis of C1GALT1 depletion in ECC-1 cells. **A**, PCR and WB analysis confirmed the efficient depletion of C1GALT1 achieved with the three shRNAs. **B**, Scatter plot proteins identified in the cell extract and secretome by LC-MS/MS after SILAC labelling. Ratios among proteins in the heavy and light versions were used as fold-change. The x-axis is log_2_ fold change H/L for the forward experiment; the y-axis is log_2_ fold change H/L for the reverse experiment. Vertical black lines represent 0.58-log_2_fold expression difference in the forward experiment, and horizontal black lines represent 0.58-log_2_fold expression difference in the reverse experiment. Proteins differentially expressed in the forward and reverse experiments are represented in green (upregulated) and red (downregulated). H, heavy; L, light. **C**, Venn diagram of the 227 differentially expressed proteins identified and quantified in the cell extract and secretome by LC-MS/MS analysis; with 17 proteins commonly dysregulated in both cellular components. **D**, String analysis of the 227 dysregulated proteins by C1GALT1 depletion revealed 24 different clusters of interaction containing three or more proteins and closely related to cancer development. Proteins not contained in any cluster were not represented. **E**, ssGSEA analysis of the more relevant glycosylation (35) and angiogenesis (27) related pathways associated to dysregulated proteins. A significant FDR correlation with C1GALT1 proteins expression was observed for 33 of the 63 pathways scored

First, depletion of C1GALT1 in ECC-1 cells using three different shRNAs was efficiently achieved as observed by PCR and WB (Fig. 2 A). shRNA SEQ2 was able to induce the highest silencing of C1GALT1 either at mRNA or protein level. ECC-1 stably C1GALT1 depleted cells with shRNA SEQ2 (shC1GALT1) were used to characterize their proteome in comparison to control cells (SCRAMBLE) to get a better understanding of the molecular and functional pathways altered in this cellular model of aggressive EC.

Then, *Forward* and *Reverse* SILAC experiments were performed; and whole protein extracts and the secretome of shC1GALT1 and SCRAMBLE ECC-1 cells analyzed. After metabolic labelling of shC1GALT1 and SCRAMBLE ECC-1 cells, proteins were analyzed by mass spectrometry. Following normalization and considering together *Forward* and *Reverse* experiments, 4012 and 5902 proteins were identified, with 3616 and 5208 proteins quantified from the secretome and the cell extracts, respectively (Supplementary Tables 1–2).

Proteins with a log_2_foldchange ≥ 0.58 (upregulated) or ≤-0.58 (downregulated) in shC1GALT1 cells in comparison to SCRAMBLE’s were further analyzed to elucidate the pathways altered in aggressive ECC-1 cells. Proteomic analysis of the secretomes showed 67 proteins upregulated and 77 downregulated, and 14 and 86 proteins in the cell extract of shC1GALT1 (Fig. 2B). Among them, 5 upregulated and 12 downregulated proteins were observed in common in both cellular components, which should be of further interest for the study of the disease (Fig. 2 C, Supplementary Tables 1–2).

In addition, gene ontology analysis was performed with DAVID to obtain the main cellular localizations of dysregulated proteins (p value < 0.02, Fig. S2). As expected, secretome dysregulated proteins were mainly found in the extracellular space and exosomes and involved in focal and cell-cell adhesion. On the other hand, proteins observed as altered in the cell extract analysis showed a more diverse distribution with cytoplasmic, nucleus, membrane, endoplasmic reticulum, and exosome as the top observed localizations (p value < 0.02). These results confirmed the correct spatial distribution of the dysregulated proteins found.

### Bioinformatics analysis

We performed a bioinformatic analysis to identify pathways and clusters of interaction taking together the dysregulated proteins in both compartments. The 227 unique proteins out of 244 dysregulated proteins identified from the secretome and cell extract analysis (76 upregulated and 151 downregulated proteins in both compartments) were analyzed using STRING and Reactome databases to identify altered pathways. STRING revealed 24 clusters of interaction composed of three or more interacting proteins, highlighting gene expression, cell adhesion, extracellular matrix organization and metabolic processes (Fig. 2D). Reactome analysis of dysregulated proteins in the cell extract of shC1GALT1 ECC-1 cells showed significant pathways encompassing most of these proteins, such as metabolism, gene expression, and cell death signaling (p value < 0.05, Fig. S3A). In addition, proteins dysregulated in the secretome were involved in extracellular matrix organization, signal transduction, cell-cell communication or transport (p value < 0.05, Fig. S3B).

Next, ssGSEA performed with CPTAC data scored 63 pathways, where 35 dysregulated pathways were related to glycosylation, 27 to angiogenesis and 1 to endometrial cancer. Importantly, 33 out of the 63 scored pathways displayed a significant FDR corrected correlation with C1GALT protein expression (FDR < 0.05) (Fig. 2E), demonstrating an important role of C1GALT1 in EC. Most of these correlated pathways (27/33) were related to glycosylation processes. The remaining pathways were associated with vascular remodeling, vessel maturation and angiogenesis. The two vascular remodeling related pathways were positively correlated with C1GALT1 protein expression, while pathways associated with vessel maturation and endothelial cell migration were negatively correlated.

### Validation of the dysregulation of selected proteins in shC1GALT1 ECC-1 cells

After bioinformatics analysis, nine proteins with the highest and lowest fold-change values were selected for further validation (Table 1) at mRNA and protein level.

**Table 1:** Dysregulated proteins in the cellular extract and/or the secretome of shC1GALT1 ECC-1 cells selected for validation

Protein IDs	Protein names	Gene names	Cellular component	Dysregulation
P04083	Annexin A1	ANXA1	Cell extract; Secretome	Upregulated
Q9Y5P4	Collagen type IV alpha-3-binding protein	COL4A3BP	Cell extract; Secretome	Upregulated
Q9Y6K1	DNA (cytosine-5)-methyltransferase 3 A	DNMT3A	Cell extract	Downregulated
P17931	Galectin-3	LGALS3	Cell extract	Upregulated
Q9UDY8	Mucosa-associated lymphoid tissue lymphoma translocation protein 1	MALT1	Secretome	Upregulated
Q15788	Nuclear receptor coactivator 1	NCOA1	Cell extract	Downregulated
Q8NBP7	Proprotein convertase subtilisin/kexin type 9	PCSK9	Secretome	Upregulated
P78504	Protein jagged-1	JAG1	Secretome	Upregulated
P19447	TFIIH basal transcription factor complex helicase XPB subunit	ERCC3	Cell extract	Downregulated

**Fig. 3:**
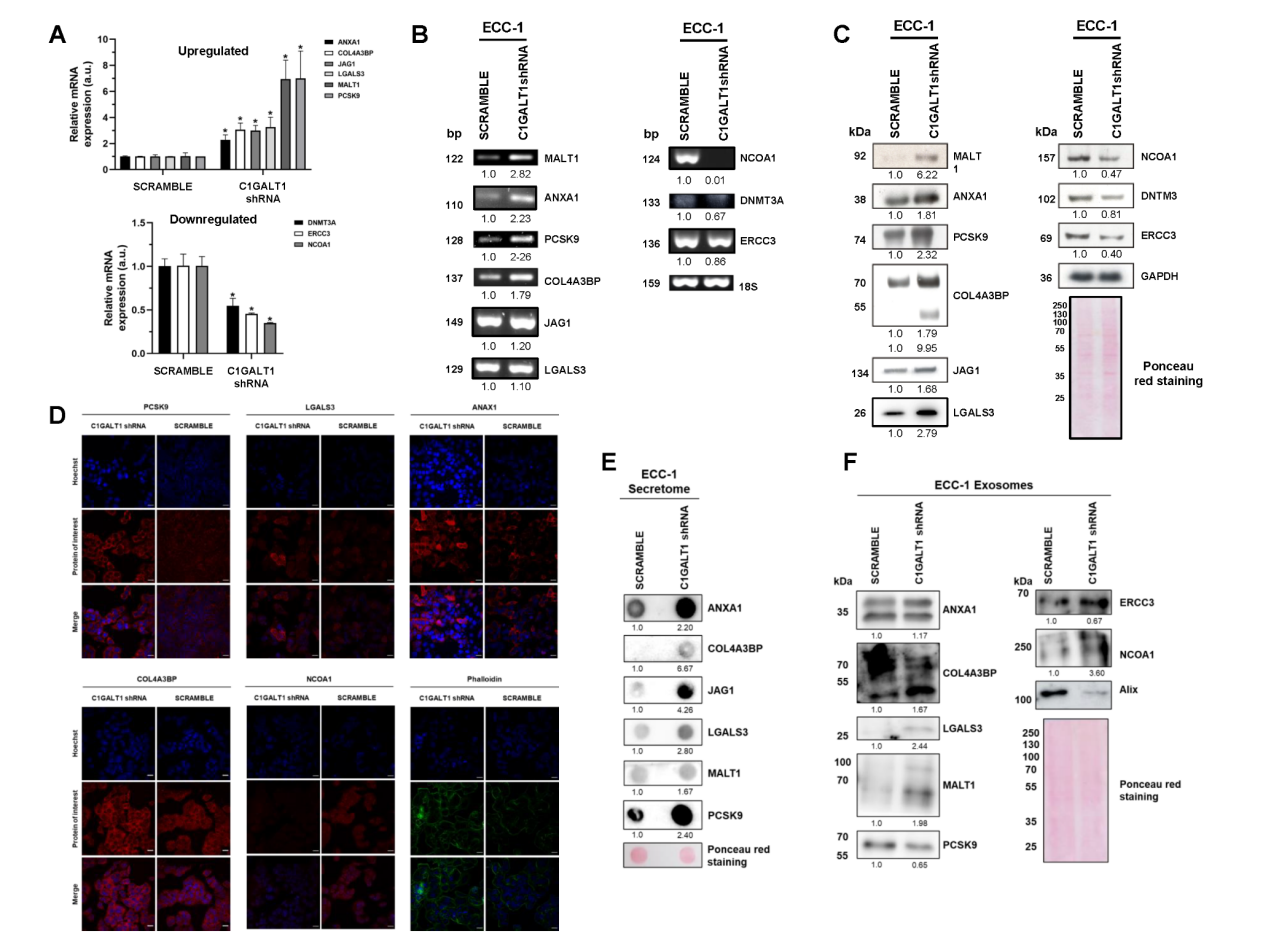
Validation of dysregulated proteins in shC1GALT1 ECC-1 cells. **A**, qPCR and **B**, PCR analysis revealed the dysregulation of selected proteins at mRNA level in shC1GALT1 cells. mRNA 18S expression levels were used for normalization. **C**, WB analysis of the whole cell extract confirmed the dysregulation of selected proteins previously observed by LC-MS/MS analysis. In addition, proteins identified as upregulated in the secretome of shC1GALT1 cells (JAG1, MALT1 and PCSK9) were also found upregulated in the cell extract. Protein levels of GAPDH were used for normalization. **D**, Immunofluorescence analysis of shC1GALT1 and SCRAMBLE ECC-1 cells confirmed the dysregulation previously observed by mass spectrometry for ANXA1, LGALS3, PCSK9, COL4A3BP and NCOA1, highlighting an expression shift for AXNA1 and PCSK9 to the cytoplasm and the plasmatic membrane, respectively, in ECC-1 C1GALT1 depleted cells. Phalloidin staining was higher in shC1GALT1 cells, suggesting a more aggressive phenotype of shC1GALT1 cells. **E**, Dot blot analysis of the secretome of shC1GALT1 and SCRAMBLE cells corroborate the dysregulation observed by LC-MS/MS for ANXA1, COL4A3BP, JAG1, LGALS3, MALT1 and PCSK9. **F**, WB analysis of exosomes secreted by ECC-1 cells showed the upregulation of ANXA1, COL4A3BP, LGALS3, MALT1, ERCC3 and NCOA1, and the downregulation of PCSK9 in shC1GALT1 ECC-1 cells’ exosomes. Alix was used as a control of extracellular vesicles. Ponceau staining was used for normalization in the secretome and exosome analysis. *, p value < 0.05

First, by semi-quantitative PCR and qPCR, we found diminished mRNA levels of DNMT3A, ERCC3 and NCOA1 in shC1GALT1 in comparison with SCRAMBLE ECC-1 cells; whereas mRNA levels of ANXA1, COL4A3BP, JAG1, LGALS3, MALT1, and PCSK9 were upregulated in C1GALT1 depleted cells (Fig. 3 A,B). In addition, the total protein content of shC1GALT1 and SCRAMBLE ECC-1 cells analyzed by WB showed lower levels of NCOA1, DNMT3A and ERCC3 and higher levels of MALT1, ANXA1, PCSK9, COL4A3BP, JAG1 and LGALS3 in shC1GALT1 cells (Fig. 3 C). Interestingly, a second band for COL4A3BP (lower than 55 kDa) was only observed in the shC1GALT1 cell extracts, which might represent the aberrant glycosylated form of COL4A3BP. Importantly, these results were in concordance with the previous mass spectrometry data, confirming their dysregulation in C1GALT1 depleted ECC-1 EC cells, and suggesting a potential role of these proteins in the development of aggressive ECs.

Moreover, we analyzed shC1GALT1 and SCRAMBLE ECC-1 cells by IF to investigate for alterations in the localization of selected proteins under native conditions. ANXA1, LGALS3, PCSK9 and COL4A3BP were overexpressed, whereas NCOA1 was downregulated in shC1GALT1 cells (Fig. 3D), as previously observed. In addition, we observed differences in the localization of ANXA1 and PCSK9 when silencing C1GALT1. ANXA1 expression was increased in the cytoplasm of shC1GALT1 cells, whereas PCSK9 expression shifted from a more cytoplasmic localization to the plasmatic membrane after C1GALT1 depletion. Furthermore, phalloidin staining was performed to analyze the expression of actin filaments (F-actin) in ECC-1. A higher phalloidin staining was observed in shC1GALT1 cells, suggesting that ECC-1 cells are more dynamic, and confirming the aggressive phenotype of ECC-1 cells after C1GALT1 depletion.

Next, levels of dysregulated proteins in the conditioned medium (secretome) of shC1GALT1 and SCRAMBLE ECC-1 cells were analyzed by dot blot. All upregulated proteins in the secretome of depleted cells by LC/MS-MS were also observed upregulated in shC1GALT1 ECC-1 cells, confirming previous proteomics data (Fig. 3E).

Finally, we further analyzed the protein content of exosomes secreted by these cell lines to investigate the potential role of selected proteins in cell communication and metastasis. As previously observed in the secretome and cell extract, ANXA1, COL4A3BP, LGALS3 and MALT1 were found upregulated in exosomes secreted by shC1GALT1 cells. Interestingly, ERCC3 and NCOA1 were overexpressed and PCSK9 levels were decreased in exosomes secreted by silencing cells, in contrast to the dysregulation observed in the cell extracts and secretome of shC1GALT1 cells (Fig. 3 F).

### C1GALT1 depletion increase the aggressiveness of EC cells

Since results pointed out to a more aggressive phenotype upon C1GALT1 depletion, we performed loss-of-function in vitro cell-based assays to confirm the involvement of C1GALT1 low expression in the increase of tumorigenic and metastatic properties of ECC-1 cells. To that end, stable depleted shC1GALT1 ECC-1 cells were used to survey for changes in adhesion, invasion, wound healing, proliferation, colony formation and angiogenesis in comparison to SCRAMBLE control ECC-1 cells.

**Fig. 4:**
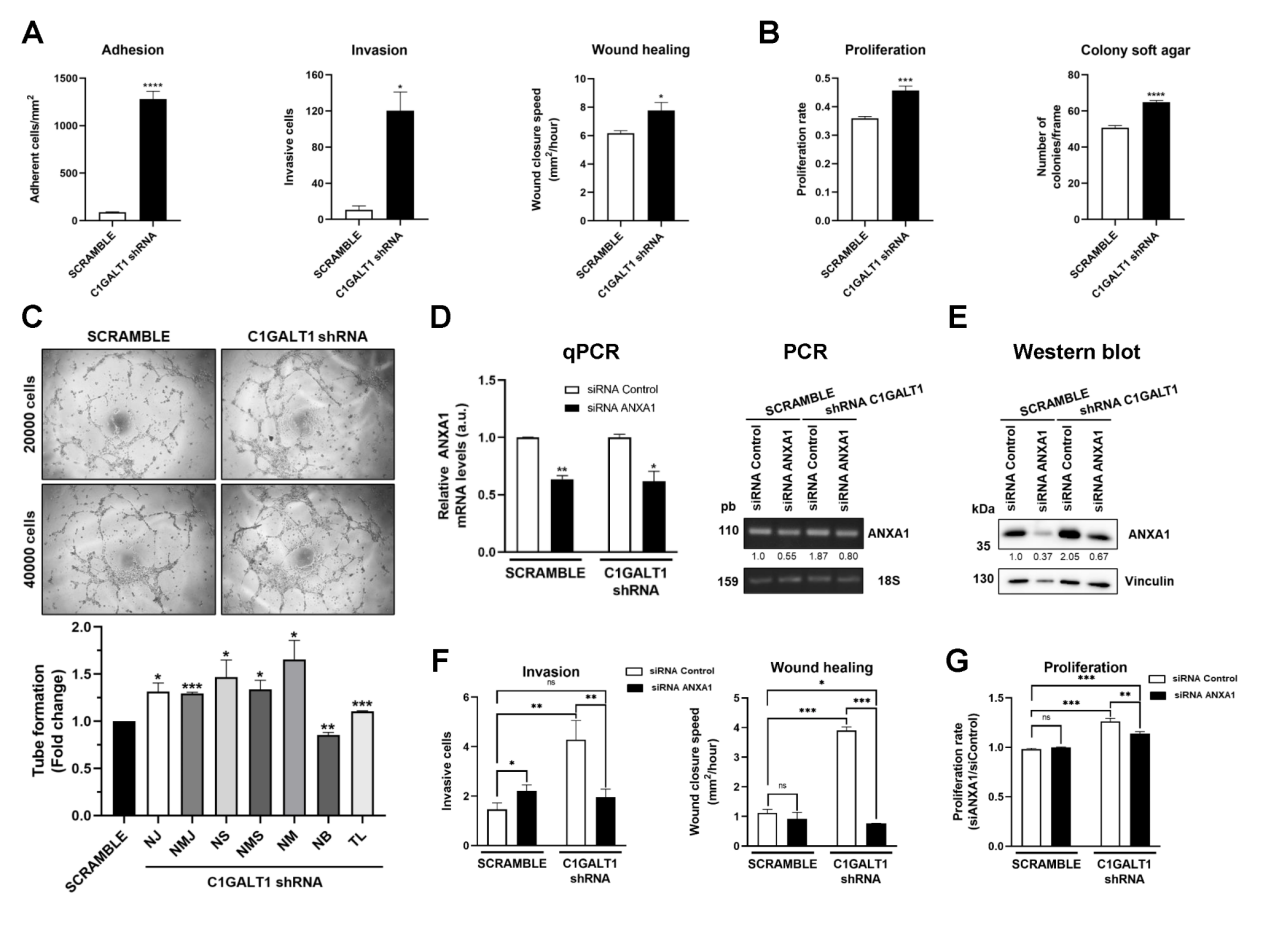
In vitro cell-based assays confirmed the association of C1GALT1 depletion with aggressive phenotypes of endometrial cancer cells. Loss-of-function assays revealed statistically significant higher **A**, metastatic and **B**, tumorigenic properties of shC1GALT1 ECC-1 cells in comparison with SCRAMBLE cells. A higher significant adhesion, invasion, close of wound healing assays, proliferation and colony soft agar formation was observed upon C1GALT1 depletion. **C**, shC1GALT1 ECC-1 cells showed higher tube formation ability than SCRAMBLE cells, with statistically significant larger number of junctions, segments, meshes and branches, and longer tubes. NJ, number of junctions; NMJ, number of master junctions; NS, number of segments; NMS, number of master segments; NM, number of meshed; NB, number of branches; TL, total length. Transient silencing of ANXA1 was assessed after shC1GALT1 and SCRAMBLE ECC-1 cells transfection with siRNA ANXA1 **D**, at mRNA level by PCR and qPCR and **E**, at protein level. For the qPCR, ANXA1 levels were related to ANXA1 in siRNA Control SCRAMBLE or shC1GALT1 levels, respectively. For the PCR and WB, ANXA1 levels were related to ANXA1 levels in SCRAMBLE siControl ECC-1 cells. 18S and vinculin were used as controls of the assays and for normalization of mRNA and protein levels, respectively. **F**, ANXA1 transient silencing significantly reduced the invasive and wound closure properties of shC1GALT1 ECC-1 cells in contrast to SCRAMBLE cells. **G**, The proliferative capacity of shC1GALT1 ECC-1 cells was significantly reversed after transient silencing of ECC-1 cells with ANXA1 siRNA, whereas SCRAMBLE cells were mostly unaffected. **F, G** Data are normalized regarding siControl SCRAMBLE ECC-1 cells. *, p value < 0.05; **, p value < 0.001; ***, p value < 0.0001; ****, p value < 0.00001

First, shC1GALT1 cells possessed a statistically significant higher adhesion and invasion capacity, and a 1.25-fold increase in their migratory ability (Fig. 4 A, S4A). Moreover, shC1GALT1 cells showed a statistically significant higher proliferation and a slight significant increase in their colony-forming ability in comparison with SCRAMBLE cells (Fig. 4B, S4B). Furthermore, tube formation assays revealed a notable higher angiogenesis ability of shC1GALT1 ECC-1 cells, provoking a significant larger number of junctions, segments, meshes and branches, and longer tubes than SCRAMBLE cells (Fig. 4 C).

These results confirmed the in vitro association of C1GALT1 with dysregulation of vascular remodeling, vessel maturation and endothelial cell migration, already shown by ssGSEA using EC samples in this paper. Collectively, we demonstrated the association of low C1GALT1 expression with an increase in tumorigenic and metastatic properties of ECC-1 cells, and thus, with a more aggressive phenotype of EC, confirming IHC and CPTAC-UCEC data.

### Correlation of C1GALT1 and ANXA1 protein expression and biological function

Accumulated evidences indicate that the ubiquitous 37-kDa ANXA1 protein dysregulation is associated with tumor development, progression, and invasion in different cancer types [43, 44]. Therefore, we hypothesized that the upregulation of ANXA1 upon C1GALT1 depletion could be associated to the increase in proliferation, invasion and migration of ECC-1 endometrial cancer cells. To address this question, we transiently depleted ANXA1 using a specific siRNA in comparison to a siRNA control on the stably transfected shC1GALT1 and SCRAMBLE ECC-1 cells to survey for alterations in their tumorigenic and metastatic properties.

First, ANXA1 depletion by transient silencing was efficiently achieved as observed by PCR, qPCR and WB analyses either on shC1GALT1 or SCRAMBLE ECC-1 cells (Fig. 4D-E). Then, we analyzed the effects on proliferation, migration and invasion. Regarding the effect of ANXA1 silencing on metastatic properties, shC1GALT1 ECC-1 were more affected than SCRAMBLE ECC-1 cells. ANXA1-silenced shC1GALT1 ECC-1 cell lines were significantly less invasive and closed the wound at a significantly slower speed than their control cells transfected with siRNA control (*p* value < 0.05), whereas no effect on wound closure speed was observed in ANXA1-silenced SCRAMBLE ECC-1 cells in comparison with their control cells (Fig. 4 F, S4C). Importantly, shC1GALT1 ECC-1 cells behave similarly than SCRAMBLE ECC-1 control cells upon ANXA1 depletion, regarding invasive and wound closure speed properties. In contrast, SCRAMBLE cells upon ANXA1 depletion increased their invasive properties about 1.5-fold.

In the proliferation assays ANXA1-silenced shC1GALT1 cells showed significant less proliferation than control cells (*p* value < 0.05). Indeed, ANXA1-silenced shC1GALT1 ECC-1 cells showed a 45% reduced proliferation regarding C1GALT1 depletion-induced proliferation (Fig. 4G). Besides, SCRAMBLE control ECC-1 cells transfected with the control siRNA and ANXA1 siRNA behave similarly (Fig. 4G).

These results demonstrate that ANXA1 silencing preferentially affected shC1GALT1 ECC-1 cells to a greater extent than SCRAMBLE cells, mostly reversing the observed induction of proliferation, migration and invasion properties upon C1GALT1 depletion. Besides, although more molecules dysregulated by the effect of C1GALT1 depletion will have an effect on tumorigenic and metastatic properties of ECC-1 cells, our results supported that effects on proliferation, migration and invasion properties were associated to the upregulation of ANXA1 as a consequence of the absence of C1GALT1. Collectively, these results correlate C1GALT1 and ANXA1 protein expression with tumorigenic and metastatic properties of endometrial cancer ECC-1 cells.

### Relevance of C1GALT1 dysregulation in EC patients

Finally, since LGALS3 (Galectin-3) has been shown to interact with O-glycans in the mucosal epithelium [30], and considering its overexpression observed by proteomics and further confirmed by PCR and WB analyses upon depletion of C1GALT1, we focused on the role of LGALS3 in EC by IHC.

**Fig. 5:**
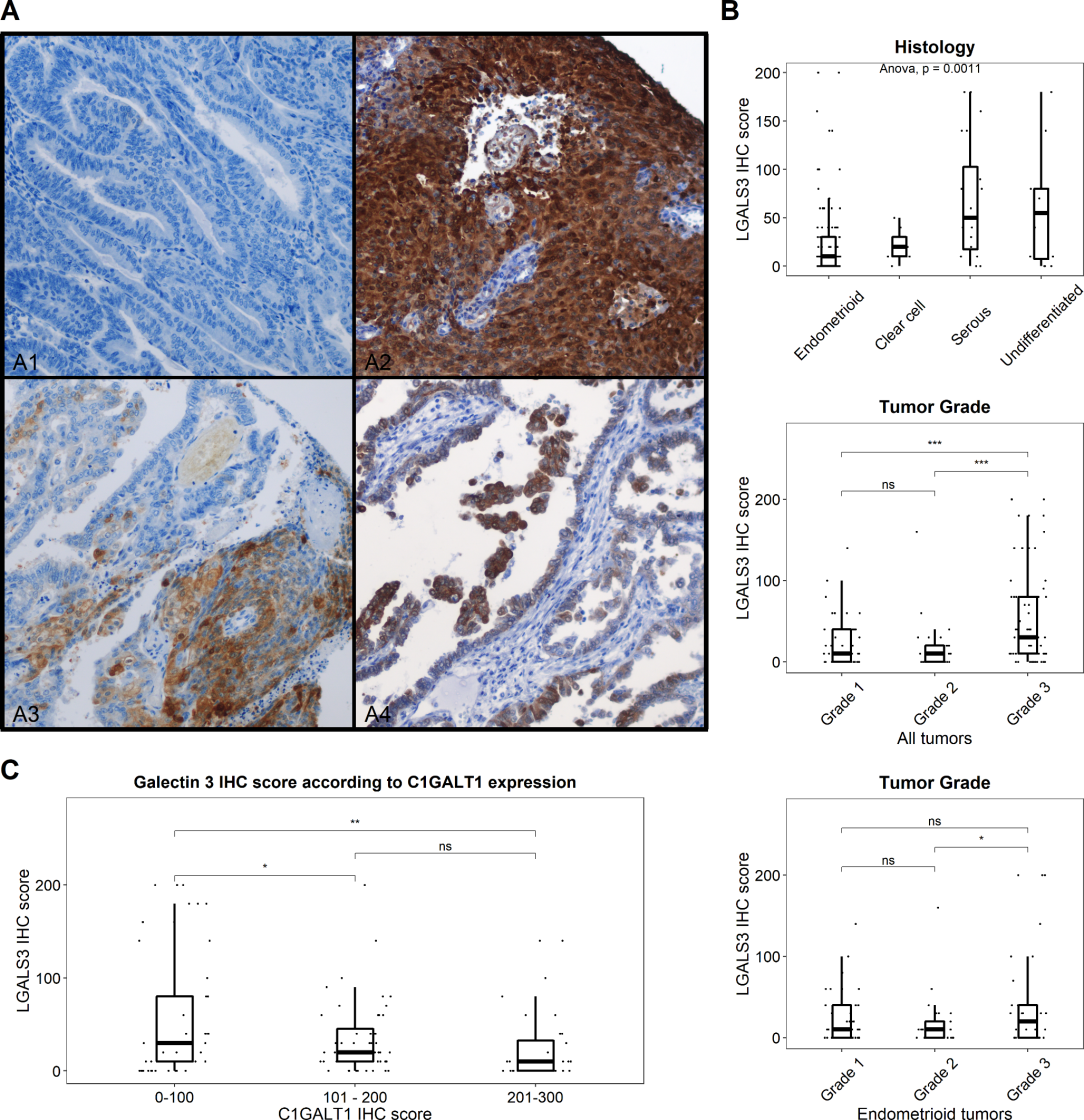
Analysis of LGALS3 expression in endometrial cancer according to the expression of C1GALT1. **A**, Representative IHC images of LGALS3 staining. A1 Negative LGALS3 expressing tumor. A2 Diffuse LGALS3 expression. A3 and A4 patchy LGALS3 expression. **B**, Immunohistochemistry analysis of 151 EC tissue samples showed a clear significant upregulation of LGALS3 expression in parallel to the aggressiveness of EC either according to histologic subtype, EC grade or tumor grade in endometrioid cancer samples. **C**, Correlation analysis of the expression of LGALS3 and C1GALT1 showed a significant negative correlation in tumor samples, demonstrating both the downregulation of C1GALT1 expression in parallel to the upregulation of LGALS3 in aggressive EC. *, p value < 0.05; **, p value < 0.001; ***, p value < 0.0001

A total of 151 out of 178 cores from 79 EC patients were considered adequate for LGALS3 expression assessment by IHC. Morphologic assessment of LGALS3 IHC staining characteristics revealed different staining patterns (Fig. 5 A). Out of the 151 evaluable cores, 45 (29.8%) showed absent LGALS3 expression. LGALS3 positive samples showed variable and low intensity protein expression (mean positive tumor cells per sample = 35.4%). A small subset of cores (13/151, 8.6%) demonstrated diffuse (> 90% positive tumor cells per sample) staining. LGALS3 score varied across histologic types, with serous and undifferentiated tumors displaying the highest protein expression (median IHC LGALS3 score = 10, 20, 50 and 55 for endometrioid, clear cell, serous and undifferentiated tumor types, respectively) (Fig. 5B). Interestingly, the aggressive histologic variants (clear cell, serous and undifferentiated) showed higher LGALS3 IHC scores than endometrioid variants (p value < 0.001). In addition, LGALS3 expression positively correlated with tumor grade. High grade tumors (G3) displayed higher protein expression (median LGALS3 IHC score = 30) compared to low grade tumors (median LGALS3 IHC score for G1/G2 tumors = 10). This finding was independent of histologic type, as similar results were observed when analyzing endometrioid tumors.

Finally, we interrogated the correlation between the expression levels of LGALS3 and C1GALT1. Importantly, a negative correlation between C1GALT1 and LGALS3 was observed in EC samples, with the highest LGALS3 expression levels occurring in cases showing the lowest expression of C1GALT1 (p value < 0.001) (Fig. 5 C). These results confirmed the antagonism between the protein expression levels of these markers, as expected according to the proteomics results.

Collectively, our results reveal that the dysregulation of O-glycosylation processes alters the protein patterns at different cellular compartments and that these changes are involved in vitro in the pathogenesis, development, and progression of the disease; as well as in EC samples.

## Discussion

O-glycosylation has emerged as a main regulator of cancer due to its role in tumor development and progression [45]. Truncated O-glycans can contribute to enhance oncogenic features in cancer cells. Anomalous O-glycosylation of cell surface receptors such as growth factors or death receptors can alter ligand binding as well as receptor trafficking and endocytosis leading to hyperproliferation [46]. In addition to the important role of O-glycosylation in cancer intracellular signaling, glycoproteins are also involved in cell-cell interactions and communication in the extracellular microenvironment. Thus, it has been shown that truncated O-glycans can induce tissue architecture loss, disturbance of basement membrane adhesion and invasive growth [47]. Moreover, O-glycosylation modification can influence tumor immune surveillance [12].

Altered O-glycosylation is observed in epithelial cancers and is frequently linked to poor prognosis. Aberrant O-glycosylation can be due to glycosyltransferase expression dysregulation. C1GALT1 is a key glycosyltransferase in the O-glycosylation process synthetizing the T antigen to which branched O-glycans are added. Elevated expression of this enzyme has been associated with tumor progression in lung adenocarcinoma and gastric cancer [48, 49]. On the contrary, ablation of C1GALT1 in gastric epithelium of mice induced spontaneous gastritis and gastric cancer and Tn antigen expression was related to tumor progression in gastric cancer patients [50]. Likewise, pancreas-specific C1GALT1 disruption originated O-glycosylation truncation that promoted early metastasis and stimulated a more aggressive pancreatic ductal adenocarcinoma phenotype [51]. Here, we focused the study on the analysis of the implication of low expression of C1GALT1 observed in this report in endometrial cancer, which account for 75–80% of all uterine cancers [5]. To this end, C1GALT1 stably depleted ECC-1 cells were obtained and demonstrated by quantitative proteomics, bioinformatics and in vitro cell-based assays to mimic an aggressive phenotype of EC.

Quantitative proteomics analysis allowed us to identify and quantify 3616 and 5208 proteins in common in *Forward* and *Reverse* SILAC experiments in the secretome and in the cell extract of ECC-1 cells, respectively, with high sensitivity and specificity in living cells [31, 52]. Proteomics analysis was performed with the cell extract and secretome of ECC-1 cells to increase the number of dysregulated proteins quantified as well as to find proteins related with cell-cell communication and metastasis, whose expression might be altered upon C1GALT1 depletion and of great interest to elucidate mechanisms of EC development associated to O-glycosylation dysregulation. More than 200 proteins were dysregulated in both analysis, and nine were selected for validation. In addition, in vitro loss-of-function assays using shC1GALT1 and SCRAMBLE ECC-1 cells confirmed the more tumorigenic and aggressive phenotype of ECC-1 cells due to C1GALT1 depletion, with a higher invasive, proliferation, migration, angiogenic and colony formation ability in comparison to SCRAMBLE cells, and thus the association of dysregulated proteins upon C1GALT1 depletion to EC aggressiveness and progression.

The nine proteins selected for validation were the most dysregulated proteins between SCRAMBLE and shC1GALT1 ECC-1 cells, and had been previously related to EC or other cancers. MALT1 (Mucosa-associated lymphoid tissue lymphoma translocation protein 1) had been identified as a key protein in the pathogenesis of different hematological and solid tumors, such as lung or prostate cancers [53, 54]. Due to its association to different signaling pathways, as the EGFR induced NF-κB activation, MALT1 has been described as an essential protein for tumor progression in preclinical models. Although high mRNA levels of MALT1 had been previously found in endometrioid EC by cDNA microarrays [55], this protein had not been previously associated to EC aggressiveness. Here, we found MALT1 overexpressed in more aggressive shC1GALT1 ECC-1’s cells, secretome and exosomes, highlighting a potential role not only in EC progression but also in metastasis. In addition, several studies propose MALT1 as a potential therapeutic target [54, 56–58], and thus, MALT1 would be suggested as an engaging candidate for the treatment of aggressive ECs lacking in the expression of C1GALT1. Regarding PCSK9 (Proprotein convertase subtilisin/kexin type 9), previous studies described an association among PCSK9 overexpression and incidence and progression of different cancers but EC. Apart from cholesterol metabolism regulation, PCSK9 promotes evasion of immune system and inhibition of inflammation in cancer [59, 60], which is in concordance with the increased plasmatic membrane expression of PCSK9 here observed by IF. Interestingly, PCSK9 inhibition is being explored as a new cancer therapy to increase the response of tumors to checkpoint therapies, which might be also applied to the treatment of aggressive ECs overexpressing PSCK9 [61, 62].

JAG1 (Jagged 1), a ligand of the Notch signaling pathway, has been associated with EC carcinogenesis through the control of multiple cellular processes. It has been described a dual role for the Notch receptor (Notch 1–4)-ligand (JAG1, JAG2, DLL1, DLL3 and DLL4) pathways as oncogenic or tumor suppressors [63–65]. Interestingly, recent studies highlight the potential role as proangiogenic regulator of the Notch-JAG1 pathway, antagonizing the Notch-DLL4 signaling. These findings pointed out to an enhanced of the DLL4-Notch pathway due to Notch glycosylation, which weakened JAG1 signaling [66–69]. In our work, we found a higher angiogenic potential for C1GALT1 depleted ECC-1 cells, which might be related to the alterations of glycosylation pathways induced by C1GALT1 silencing and JAG1 overexpression. In addition, Annexin A1 (ANXA1) is a ubiquitous protein involved in different processes, as calcium signaling, inflammation, cell proliferation, apoptosis or tumor progression, and which upregulation has been associated with tumor proliferation in breast, bladder, endometrial, colorectal, lung and pancreatic cancer, and melanoma [70–74]. Previous works described ANXA1 upregulated in the secretome and exosomes secreted by cancer cells [75], which has been associated with a major potential role of ANXA1 in promoting cancer angiogenesis, proliferation, invasion and metastatic properties of cancer cells [43, 44]. Here, ANXA1 was demonstrated to be upregulated in the cell extract, secretome and exosomes of C1GALT1 depleted cells, which might also be involved in their higher angiogenic potential together with JAG1 signaling and in progression and metastatic potential of EC cells. Besides, ANXA1 was here found by IF increased in the cytoplasm of shC1GALT1 cells, suggesting a significant role of ANXA1 signaling in EC aggressiveness. More importantly, we also here demonstrate that the increase on proliferation, invasion and migration of ECC-1 endometrial cancer cells upon depletion of C1GALT1 was associated to the overexpression of ANXA1. We observed here that these effects were mostly reverted upon transient ANXA1 silencing using a specific siRNA. Therefore, although other players would also be responsible of these effects, we were able to demonstrate the causal correlation between C1GALT1 and ANXA1 protein expression with tumorigenic and metastatic properties of endometrial cancer cells.

Moreover, COL4A3BP (Ceramide transfer protein or CERT) is a lipid transfer protein that transport ceramide, a pro-apoptotic signal molecule, from the endoplasmic reticulum (ER) to the Golgi apparatus [76], which has been found upregulated in different cancers, such as ovarian cancer [77]. Although COL4A3BP dysregulation has not been previously associated with EC, this protein has been proposed as an interesting therapeutic target for chemotherapy-resistant cancers due to the effect of COL4A3BP downregulation in promoting endoplasmic reticulum stress [77, 78]. Here, COL4A3BP was found upregulated in the cell extract, secretome and exosomes of C1GALT1 depleted cells, suggesting its potential role in EC development, and as therapeutic target of aggressive ECs. In addition, a second isoform of COL4A3BP probably corresponding to an aberrant glycosylated form of the protein was found increased in the cell extract and exosomes of shC1GALT1 cells. NCOA1 (Nuclear receptor coactivator 1) is a hormone-dependent regulator of gene expression associated to different cancers, such as breast and esophageal carcinomas [79]. Although endometrioid ECC-1 cells are characterized to maintain NCOA1 expression [29], as well as other androgen and estrogen receptors, its role in EC has not been elucidated yet. Because aggressive type II ECs are not associated with estrogen excess, NCOA1 downregulation might also validate the aggressive potential of endometrioid cells upon C1GALT1 depletion. On the contrary, high levels of NCOA1 were found in exosomes, which postulate the role of this protein in the establishment of the metastatic niche. In addition, DNMT3A (DNA (cytosine-5)-methyltransferase 3 A), a transcriptional corepressor in endometrioid cancers, is characterized to be overexpressed in endometrioid cancers [80, 81]. However, opposite DNA methylation patterns (associated with a higher mutation rate in aggressive ECs) were found between type I and type II ECs, thus confirming the more aggressive phenotype of ECC-1 cells induced upon C1GALT1 depletion [82]. These data suggest that NCOA1 and DNMT3A levels might be used as biomarkers of EC malignancy. In contrast, dysregulation of ERCC3 (Excision repair cross-complementation group 3) levels has not been previously associated to EC, although different studies highlight the suppressor role of ERCC3 silencing in different cancers, as pancreatic or liver carcinomas [83, 84].

Finally, LGALS3 (Galectin-3) is a protein that interacts with glycoproteins from the extracellular matrix in a galactose-dependent manner, favoring cell interactions, or with cytosolic or nuclear targets in a glycosylation independent manner [46]. Importantly, LGALS3 has been reported as EC marker [85–87], and as unfavorable marker for overall survival [73]. Here, we have found that the LGALS3 upregulation occurred in parallel to the downregulation of C1GALT1 both in vitro and in vivo in tumor tissue, with a significant negative correlation between them. Therefore, it can be suggested that LGALS3 upregulation in aggressive EC was a consequence of the downregulation of O-glycosylation in proteins from the extracellular matrix, which avoids LGALS3 interaction in the extracellular matrix of EC tumors, and as a compensatory effect an increase and release of LGALS3 should be produced.

In conclusion, quantitative proteomics of a well-characterized cellular model, where upon C1GALT1 depletion a more aggressive phenotype was induced, allow for the identification of proteins dysregulated in aggressive ECs and related pathways that might be of interest for a better understanding of the mechanisms undergoing EC pathogenesis related to O-glycosylation. Proteins here identified by proteomics as dysregulated in aggressive EC were validated by orthogonal techniques using cellular models of non-aggressive (SCRAMBLE) and aggressive (shC1GALT1 ECC-1) endometrial cancer. In addition, a causal effect on the tumorigenic and metastatic properties of C1GALT1 depleted ECC-1 cells through ANXA1 was found, where ANXA1 transient depletion provoked a reversion on proliferation, invasion and migration properties induced because of the absence of C1GALT1. However, as potential limitations of the study, further research would be needed using animal models and a larger cohort of tissue samples from EC patients from different hospitals to further confirm the association between C1GALT1 depletion and EC aggressiveness and protein dysregulation. In addition, the nine most dysregulated proteins here identified by proteomics and validated by orthogonal techniques could be interesting to investigate their role in the pathogenesis and development of aggressive ECs. LGALS3 and C1GALT1 could be used as specific biomarkers of aggressive ECs, which would allow for the earlier treatment of patients with this phenotype associated to more aggressive ECs with higher metastatic potential. Furthermore, the diagnostic value of proteins here identified as upregulated in the secretome of aggressive ECC-1 could be investigated by ELISA to elucidate the potential role of ANXA1, COL4A3BP, JAG1, MALT1, LGALS3 and PCSK9 as blood-based biomarkers of the disease.

## Electronic supplementary material

Below is the link to the electronic supplementary material.

Supplementary Material 1

Supplementary Material 2

Supplementary Material 3

Supplementary Material 4

Supplementary Material 5

Supplementary Material 6

Supplementary Material 7

Supplementary Material 8

## Data Availability

All data sets generated during the study are available in the Supplementary tables. The mass spectrometry proteomics data have been deposited to the ProteomeXchange Consortium via the PRIDE partner repository with the dataset identifier PXD032271.

## References

[1] M. Koskas, F. Amant, M.R. Mirza, C.L. Creutzberg, Int. J. Gynaecol. Obstet. **155**(Suppl 1), 45–60 (2021). 10.1002/ijgo.13866 34669196 10.1002/ijgo.13866 PMC9297903

[2] F. Amant, P. Moerman, P. Neven, D. Timmerman, E. Van Limbergen, I. Vergote, Lancet **366**, 491–505 (2005). 10.1016/S0140-6736(05)67063-8 16084259 10.1016/S0140-6736(05)67063-8

[3] J.I. Sorosky, Obstet. Gynecol. **120**, 383–397 (2012). 10.1097/AOG.0b013e3182605bf1 22825101 10.1097/AOG.0b013e3182605bf1

[4] I.B. Engelsen, L.A. Akslen, H.B. Salvesen, APMIS **117**, 693–707 (2009). 10.1111/j.1600-0463.2009.02467.x 19775337 10.1111/j.1600-0463.2009.02467.x

[5] K. Passarello, S. Kurian, V. Villanueva, Semin Oncol. Nurs. **35**, 157–165 (2019). 10.1016/j.soncn.2019.02.002 30867105 10.1016/j.soncn.2019.02.002

[6] M. Saleh, M. Virarkar, P. Bhosale, S. El Sherif, S. Javadi, S.C. Faria, J. Comput. Assist. Tomogr **44**, 714–729 (2020). 10.1097/RCT.0000000000001025 32842057 10.1097/RCT.0000000000001025

[7] R.A. Soslow, C. Tornos, K.J. Park, A. Malpica, X. Matias-Guiu, E. Oliva, V. Parkash, J. Carlson, W.G. McCluggage, C.B. Gilks, Int. J. Gynecol. Pathol. **38**(Suppl 1), S64–S74 (2019). 10.1097/PGP.0000000000000518 30550484 10.1097/PGP.0000000000000518 PMC6295928

[8] I. Ruz-Caracuel, J.L. Ramon-Patino, A. Lopez-Janeiro, L. Yebenes, A. Berjon, A. Hernandez, A. Gallego, V. Heredia-Soto, M. Mendiola, A. Redondo, A. Pelaez-Garcia, D. Hardisson, Cancers (Basel) **11**, (2019) 10.3390/cancers11121845 10.3390/cancers11121845 PMC6966575 31766622

[9] R. Gupta, F. Leon, S. Rauth, S.K. Batra, M.P. Ponnusamy, Cells **9**, (2020) 10.3390/cells9020446 10.3390/cells9020446 PMC7072808 32075174

[10] J. Munkley, D.J. Elliott, Oncotarget **7**, 35478–35489 (2016). 10.18632/oncotarget.8155 27007155 10.18632/oncotarget.8155 PMC5085245

[11] A.M. Martins, C.C. Ramos, D. Freitas, C.A. Reis, Cells **10**, (2021) 10.3390/cells10010109 10.3390/cells10010109 PMC7827205 33430152

[12] C. Fu, H. Zhao, Y. Wang, H. Cai, Y. Xiao, Y. Zeng, H. Chen, HLA **88**, 275–286 (2016) 10.1111/tan.12900 10.1111/tan.12900 27679419

[13] T. Kurita, T.N. Thi, C. Koi, M. Murakami, S. Kagami, H. Izumi, T. Hachisuga, Anticancer Res. **37**, 3905–3910 (2017). 10.21873/anticanres.11772 28668893 10.21873/anticanres.11772

[14] T.T. Nguyen, T. Kurita, C. Koi, M. Murakami, S. Kagami, T. Hachisuga, H. Masanori, Y. Morimoto, H. Izumi, Am. J. Cancer Res. **7**, 1188–1197 (2017) 28560066 PMC5446483

[15] G.M. Trinca, C.R. Hagan, J. Bioenerg Biomembr. **50**, 199–204 (2018). 10.1007/s10863-017-9730-z 29127647 10.1007/s10863-017-9730-z PMC5945348

[16] A. Krzeslak, K. Wojcik-Krowiranda, E. Forma, A. Bienkiewicz, M. Brys, Ginekol. Pol. **83**, 22–26 (2012) 22384635

[17] A. Guzman-Aranguez, F. Mantelli, P. Argueso, Invest. Ophthalmol. Vis. Sci. **50**, 4581–4587 (2009). 10.1167/iovs.09-3563 19407012 10.1167/iovs.09-3563 PMC2751810

[18] G.F. Springer, P.R. Desai, I. Banatwala, J. Natl. Cancer Inst. **54**, 335–339 (1975) 163330

[19] N.Y. Lin, S.T. Chen, H.L. Chang, M.Y. Lu, Y.L. Yang, S.W. Chou, D.T. Lin, K.H. Lin, S.T. Jou, W.M. Hsu, M.C. Huang, H.H. Chang, Oncogenesis **11**, 8 (2022) 10.1038/s41389-022-00383-w 10.1038/s41389-022-00383-w PMC8847342 35169131

[20] I. Ruz-Caracuel, A. Lopez-Janeiro, V. Heredia-Soto, J.L. Ramon-Patino, L. Yebenes, A. Berjon, A. Hernandez, A. Gallego, P. Ruiz, A. Redondo, A. Pelaez-Garcia, M. Mendiola, D. Hardisson, Virchows Arch. **479**, 1167–1176 (2021). 10.1007/s00428-021-03176-5 34420090 10.1007/s00428-021-03176-5 PMC8724178

[21] Y. Dou, E.A. Kawaler, D. Cui Zhou, M.A. Gritsenko, C. Huang, L. Blumenberg, A. Karpova, V.A. Petyuk, S.R. Savage, S. Satpathy, W. Liu, Y. Wu, C.F. Tsai, B. Wen, Z. Li, S. Cao, J. Moon, Z. Shi, M. Cornwell, M.A. Wyczalkowski, R.K. Chu, S. Vasaikar, H. Zhou, Q. Gao, R.J. Moore, K. Li, S. Sethuraman, M.E. Monroe, R. Zhao, D. Heiman, K. Krug, K. Clauser, R. Kothadia, Y. Maruvka, A.R. Pico, A.E. Oliphant, E.L. Hoskins, S.L. Pugh, S.J.I. Beecroft, D.W. Adams, J.C. Jarman, A. Kong, H.Y. Chang, B. Reva, Y. Liao, D. Rykunov, A. Colaprico, X.S. Chen, A. Czekanski, M. Jedryka, R. Matkowski, M. Wiznerowicz, T. Hiltke, E. Boja, C.R. Kinsinger, M. Mesri, A.I. Robles, H. Rodriguez, D. Mutch, K. Fuh, M.J. Ellis, D. DeLair, M. Thiagarajan, D.R. Mani, G. Getz, M. Noble, A.I. Nesvizhskii, P. Wang, M.L. Anderson, D.A. Levine, R.D. Smith, S.H. Payne, K.V. Ruggles, K.D. Rodland, L. Ding, B. Zhang, T. Liu, D. Fenyo and C. Clinical Proteomic Tumor Analysis, Cell **180**, 729–748 e726 (2020) 10.1016/j.cell.2020.01.026

[22] S.V. Vasaikar, P. Straub, J. Wang, B. Zhang, Nucleic Acids Res **46**, D956-D963 (2018) 10.1093/nar/gkx1090 10.1093/nar/gkx1090 PMC5753188 29136207

[23] S. Hanzelmann, R. Castelo, J. Guinney, BMC Bioinform **14**, 7 (2013). 10.1186/1471-2105-14-7 10.1186/1471-2105-14-7 PMC3618321 23323831

[24] I. Dolgalev, https://igordot.github.io/msigdbr/. Accessed 8-12 November 2021

[25] D. Szklarczyk, A.L. Gable, D. Lyon, A. Junge, S. Wyder, J. Huerta-Cepas, M. Simonovic, N.T. Doncheva, J.H. Morris, P. Bork, L.J. Jensen, C.V. Mering, Nucleic Acids Res **47**, D607–D613 (2019). 10.1093/nar/gky1131 30476243 10.1093/nar/gky1131 PMC6323986

[26] D.W. Huang, B.T. Sherman, Q. Tan, J. Kir, D. Liu, D. Bryant, Y. Guo, R. Stephens, M.W. Baseler, H.C. Lane, R.A. Lempicki, Nucleic Acids Res **35**, W169–W175 (2007). 10.1093/nar/gkm415 17576678 10.1093/nar/gkm415 PMC1933169

[27] G. Dennis Jr., B.T. Sherman, D.A. Hosack, J. Yang, W. Gao, H.C. Lane, R.A. Lempicki, Genome Biol. **4**, P3 (2003)12734009

[28] P.G. Satyaswaroop, S.S. Tabibzadeh, Cancer Res. **51**, 5661–5666 (1991) 1913685

[29] B. Mo, A.E. Vendrov, W.A. Palomino, B.R. DuPont, K.B. Apparao, B.A. Lessey, Biol. Reprod. **75**, 387–394 (2006). 10.1095/biolreprod.106.051870 16707768 10.1095/biolreprod.106.051870

[30] P. Argueso, A. Guzman-Aranguez, F. Mantelli, Z. Cao, J. Ricciuto, N. Panjwani, J. Biol. Chem. **284**, 23037–23045 (2009). 10.1074/jbc.M109.033332 19556244 10.1074/jbc.M109.033332 PMC2755710

[31] M. Mendes, A. Pelaez-Garcia, M. Lopez-Lucendo, R.A. Bartolome, E. Calvino, R. Barderas, J.I. Casal, Proteomics **17**, (2017) 10.1002/pmic.201700094 10.1002/pmic.201700094 28861940

[32] H. Nguyen, I.A. Wood, M.M. Hill, J. Integr. OMICS **2**, 80–93 (2012)

[33] A. Pelaez-Garcia, R. Barderas, R. Batlle, R. Vinas-Castells, R.A. Bartolome, S. Torres, M. Mendes, M. Lopez-Lucendo, R. Mazzolini, F. Bonilla, A. Garcia de Herreros, J.I. Casal, Mol. Cell. Proteomics **14**, 303–315 (2015). 10.1074/mcp.M114.045328 25505127 10.1074/mcp.M114.045328 PMC4350027

[34] G. Solis-Fernandez, A. Montero-Calle, J. Martinez-Useros, A. Lopez-Janeiro, V. de Los Rios, R. Sanz, J. Dziakova, E. Milagrosa, M.J. Fernandez-Acenero, A. Pelaez-Garcia, J.I. Casal, J. Hofkens, S. Rocha, R. Barderas, Cells **11**, (2022) 10.3390/cells11030447 10.3390/cells11030447 PMC8834500 35159257

[35] R. Barderas, M. Mendes, S. Torres, R.A. Bartolome, M. Lopez-Lucendo, R. Villar-Vazquez, A. Pelaez-Garcia, E. Fuente, F. Bonilla, J.I. Casal, Mol. Cell. Proteomics **12**, 1602–1620 (2013). 10.1074/mcp.M112.022848 23443137 10.1074/mcp.M112.022848 PMC3675817

[36] A. Montero-Calle et al., Engineering **7**, 19 (2021). 10.1016/j.eng.2021.04.026

[37] J.R. Wisniewski, F.Z. Gaugaz, Anal. Chem. **87**, 4110–4116 (2015). 10.1021/ac504689z 25837572 10.1021/ac504689z

[38] S. Borowicz, M. Van Scoyk, S. Avasarala, M.K. Karuppusamy Rathinam, J. Tauler, R.K. Bikkavilli, R.A. Winn, J. Vis. Exp., e51998 (2014) 10.3791/51998 10.3791/51998 PMC4353381 25408172

[39] A. Pelaez-Garcia, R. Barderas, S. Torres, P. Hernandez-Varas, J. Teixido, F. Bonilla, A.G. de Herreros, J.I. Casal, PLoS One **8**, e63695 (2013). 10.1371/journal.pone.0063695 23696849 10.1371/journal.pone.0063695 PMC3655941

[40] G. Solís-Fernández, A. Montero-Calle, M. Sanchez-Martinez, A. Pelaez-Garcia, M.J. Fernandez-Acenero, P. Pallarés, M. Alonso-Navarro, M. Mendiola, J. Hendrix, D. Hardisson, R.A. Bartolome, J. Hofkens, S. Rocha, R. Barderas, Br. J. Cancer **126**, 1604-1615 (2022) 10.1038/s41416-022-01762-1 PMC9130499 35347323

[41] M. Garranzo-Asensio, G. Solis-Fernandez, A. Montero-Calle, J.M. Garcia-Martinez, M.C. Fiuza, P. Pallares, N. Palacios-Garcia, C. Garcia-Jimenez, A. Guzman-Aranguez, R. Barderas, Diabetes **71**, 497–510 (2022). 10.2337/db20-1206 35040477 10.2337/db20-1206

[42] A. Montero-Calle, M. Gomez de Cedron, A. Quijada-Freire, G. Solis-Fernandez, V. Lopez-Alonso, I. Espinosa-Salinas, A. Pelaez-Garcia, M.J. Fernandez-Acenero, A. Ramirez de Molina and R. Barderas, Front. Oncol. **12**, 903033 (2022) 10.3389/fonc.2022.903033 35957902 10.3389/fonc.2022.903033 PMC9358964

[43] C. Guo, S. Liu, M.Z. Sun, Future Oncol. **9**, 1773–1793 (2013). 10.2217/fon.13.114 24156336 10.2217/fon.13.114

[44] Z. Boudhraa, B. Bouchon, C. Viallard, M. D’Incan, F. Degoul, Clin. Sci. (Lond) **130**, 205–220 (2016). 10.1042/CS20150415 26769657 10.1042/CS20150415

[45] J.G. Rodrigues, M. Balmana, J.A. Macedo, J. Pocas, A. Fernandes, J.C.M. de-Freitas-Junior, S.S. Pinho, J. Gomes, A. Magalhaes, C. Gomes, S. Mereiter, C.A. Reis, Cell. Immunol. **333**, 46–57 (2018). 10.1016/j.cellimm.2018.03.007 29576316 10.1016/j.cellimm.2018.03.007

[46] S.S. Pinho, C.A. Reis, Nat. Rev. Cancer **15**, 540–555 (2015). 10.1038/nrc3982 26289314 10.1038/nrc3982

[47] P. Radhakrishnan, S. Dabelsteen, F.B. Madsen, C. Francavilla, K.L. Kopp, C. Steentoft, S.Y. Vakhrushev, J.V. Olsen, L. Hansen, E.P. Bennett, A. Woetmann, G. Yin, L. Chen, H. Song, M. Bak, R.A. Hlady, S.L. Peters, R. Opavsky, C. Thode, K. Qvortrup, K.T. Schjoldager, H. Clausen, M.A. Hollingsworth, H.H. Wandall, Proc. Natl. Acad. Sci. U S A **111**, E4066–E4075 (2014). 10.1073/pnas.1406619111 25118277 10.1073/pnas.1406619111 PMC4191756

[48] X. Dong, Y. Liu, X. Deng, J. Shao, S. Tian, S. Chen, R. Huang, Z. Lin, C. Chen, L. Shen, Front. Cell. Dev. Biol. **9**, 707970 (2021). 10.3389/fcell.2021.707970 34307388 10.3389/fcell.2021.707970 PMC8292976

[49] X. Dong, C. Chen, X. Deng, Y. Liu, Q. Duan, Z. Peng, Z. Luo, L. Shen, Cell. Biosci. **11**, 166 (2021). 10.1186/s13578-021-00678-2 34452648 10.1186/s13578-021-00678-2 PMC8393437

[50] F. Liu, J. Fu, K. Bergstrom, X. Shan, J.M. McDaniel, S. McGee, X. Bai, W. Chen, L. Xia, J. Exp. Med. **217**, (2020) 10.1084/jem.20182325 10.1084/jem.20182325 PMC7037257 31645367

[51] S. Chugh, S. Barkeer, S. Rachagani, R.K. Nimmakayala, N. Perumal, R. Pothuraju, P. Atri, S. Mahapatra, I. Thapa, G.A. Talmon, L.M. Smith, X. Yu, S. Neelamegham, J. Fu, L. Xia, M.P. Ponnusamy, S.K. Batra, Gastroenterology **155**, 1608–1624 (2018). 10.1053/j.gastro.2018.08.007 30086262 10.1053/j.gastro.2018.08.007 PMC6219903

[52] X. Chen, S. Wei, Y. Ji, X. Guo, F. Yang, Proteomics **15**, 3175–3192 (2015). 10.1002/pmic.201500108 26097186 10.1002/pmic.201500108

[53] D. Pan, C. Jiang, Z. Ma, M. Blonska, M.J. You, X. Lin, Oncogene **35**, 919–928 (2016). 10.1038/onc.2015.146 25982276 10.1038/onc.2015.146 PMC4651666

[54] B. Gomez Solsona, A. Schmitt, K. Schulze-Osthoff, S. Hailfinger, Biomedicines **10**, (2022) 10.3390/biomedicines10020344 10.3390/biomedicines10020344 PMC8961791 35203553

[55] A. Yeramian, G. Moreno-Bueno, X. Dolcet, L. Catasus, M. Abal, E. Colas, J. Reventos, J. Palacios, J. Prat, and X. Matias-Guiu, Oncogene **32**, 403–413 (2013). 10.1038/onc.2012.76 10.1038/onc.2012.76 22430211

[56] D. Vucic, V.M. Dixit, J. Exp. Med. **206**, 2309–2312 (2009). 10.1084/jem.20092160 19841088 10.1084/jem.20092160 PMC2768848

[57] C. Mc Guire, P. Wieghofer, L. Elton, D. Muylaert, M. Prinz, R. Beyaert, G. van Loo, J. Immunol. **190**, 2896–2903 (2013). 10.4049/jimmunol.1201351 23401595 10.4049/jimmunol.1201351

[58] H. Tan, Y. Xie, X. Zhang, S. Wu, H. Zhao, J. Wu, W. Wang, C. Lin, Front. Mol. Biosci. **8**, 714906 (2021). 10.3389/fmolb.2021.714906 34926571 10.3389/fmolb.2021.714906 PMC8674617

[59] J. Frostegard, Expert Rev. Clin. Immunol. **18**, 67–74 (2022). 10.1080/1744666X.2022.2017281 34928185 10.1080/1744666X.2022.2017281

[60] S.Z. Zhang, X.D. Zhu, L.H. Feng, X.L. Li, X.F. Liu, H.C. Sun, Z.Y. Tang, Exp. Hematol. Oncol. **10**, 25 (2021). 10.1186/s40164-021-00218-1 33789749 10.1186/s40164-021-00218-1 PMC8011384

[61] C.R. Almeida, B.H. Ferreira, I.F. Duarte, Signal. Transduct. Target. Ther. **6**, 111 (2021). 10.1038/s41392-021-00530-6 33677469 10.1038/s41392-021-00530-6 PMC7936974

[62] X. Liu, X. Bao, M. Hu, H. Chang, M. Jiao, J. Cheng, L. Xie, Q. Huang, F. Li, C.Y. Li, Nature **588**, 693–698 (2020). 10.1038/s41586-020-2911-7 33177715 10.1038/s41586-020-2911-7 PMC7770056

[63] Y. Mitsuhashi, A. Horiuchi, T. Miyamoto, H. Kashima, A. Suzuki, T. Shiozawa, Histopathology **60**, 826–837 (2012). 10.1111/j.1365-2559.2011.04158.x 22348356 10.1111/j.1365-2559.2011.04158.x

[64] V. Jonusiene, A. Sasnauskiene, N. Lachej, D. Kanopiene, D. Dabkeviciene, S. Sasnauskiene, B. Kazbariene, J. Didziapetriene, Med. Oncol. **30**, 438 (2013). 10.1007/s12032-012-0438-y 23315219 10.1007/s12032-012-0438-y

[65] N. Lachej, V. Jonusiene, A. Mazeike, A. Sasnauskiene, D. Dabkeviciene, J. Simiene, K. Suziedelis, J. Didziapetriene, Acta Med. Litu **26**, 181–190 (2019). 10.6001/actamedica.v26i3.4148 32015673 10.6001/actamedica.v26i3.4148 PMC6992362

[66] R. Benedito, C. Roca, I. Sorensen, S. Adams, A. Gossler, M. Fruttiger, R.H. Adams, Cell **137**, 1124–1135 (2009). 10.1016/j.cell.2009.03.025 19524514 10.1016/j.cell.2009.03.025

[67] H. Yan, L. Zhu, J. Zhang, Z. Lin, Cell. Death Discov **7**, 284 (2021). 10.1038/s41420-021-00682-y 34667158 10.1038/s41420-021-00682-y PMC8526739

[68] H. Jafar-Nejad, J. Leonardi, R. Fernandez-Valdivia, Glycobiology **20**, 931–949 (2010). 10.1093/glycob/cwq053 20368670 10.1093/glycob/cwq053 PMC2912550

[69] L.T. Yang, J.T. Nichols, C. Yao, J.O. Manilay, E.A. Robey, G. Weinmaster, Mol. Biol. Cell. **16**, 927–942 (2005). 10.1091/mbc.e04-07-0614 15574878 10.1091/mbc.E04-07-0614 PMC545923

[70] P. Li, L. Li, Z. Li, S. Wang, R. Li, W. Zhao, Y. Feng, S. Huang, L. Li, H. Qiu, S. Xia, Cancer Cell. Int. **22**, 7 (2022). 10.1186/s12935-021-02427-4 34991599 10.1186/s12935-021-02427-4 PMC8740017

[71] Z. Qian, W. Fan, F. Meng, Z. Sun, G. Li, Y. Zhai, Y. Chang, C. Yang, F. Zeng, R. Chai, F. Wu, Z. Zhao, Front. Cell. Dev. Biol. **9**, 777182 (2021). 10.3389/fcell.2021.777182 34912807 10.3389/fcell.2021.777182 PMC8667664

[72] M. Manai, R. Doghri, P. Finetti, K. Mrad, R. Bouabsa, M. Manai, D. Birnbaum, F. Bertucci, L. Charfi, M. Driss, In Vivo **34**, 177–184 (2020). 10.21873/invivo.11759 31882477 10.21873/invivo.11759 PMC6984120

[73] S. Aboulouard, M. Wisztorski, M. Duhamel, P. Saudemont, T. Cardon, F. Narducci, A.S. Lemaire, F. Kobeissy, E. Leblanc, I. Fournier, M. Salzet, Cell. Rep. Med. **2**, 100318 (2021). 10.1016/j.xcrm.2021.100318 34195683 10.1016/j.xcrm.2021.100318 PMC8233695

[74] M. Oshi, Y. Tokumaru, S. Mukhopadhyay, L. Yan, R. Matsuyama, I. Endo, K. Takabe, Cells **10**, (2021) 10.3390/cells10030653 10.3390/cells10030653 PMC8000658 33804148

[75] R. Belvedere, E. Morretta, N. Novizio, S. Morello, O. Bruno, C. Brullo, A. Petrella, Biomolecules **11**, (2021) 10.3390/biom11121758 10.3390/biom11121758 PMC8699007 34944403

[76] L.H. Chung, D. Liu, X.T. Liu, Y. Qi, Int. J. Mol. Sci. **22**, (2021) 10.3390/ijms222413184 10.3390/ijms222413184 PMC8705978 34947980

[77] R.P. Rao, L. Scheffer, S.M. Srideshikan, V. Parthibane, T. Kosakowska-Cholody, M.A. Masood, K. Nagashima, P. Gudla, S. Lockett, U. Acharya, J.K. Acharya, PLoS One **9**, e92142 (2014). 10.1371/journal.pone.0092142 24642596 10.1371/journal.pone.0092142 PMC3958450

[78] C. Swanton, M. Marani, O. Pardo, P.H. Warne, G. Kelly, E. Sahai, F. Elustondo, J. Chang, J. Temple, A.A. Ahmed, J.D. Brenton, J. Downward, B. Nicke, Cancer Cell. **11**, 498–512 (2007). 10.1016/j.ccr.2007.04.011 17560332 10.1016/j.ccr.2007.04.011

[79] L. Wang, W. Li, K. Li, Y. Guo, D. Liu, Z. Yao, X. Lin, S. Li, Z. Jiang, Q. Liu, Y. Jiang, B. Zhang, L. Chen, F. Zhou, H. Ren, D. Lin, D. Zhang, S.J. Yeung, H. Zhang, Cancer Med. **7**, 5205–5216 (2018). 10.1002/cam4.1786 30270520 10.1002/cam4.1786 PMC6198200

[80] D. He, X. Wang, Y. Zhang, J. Zhao, R. Han, Y. Dong, Chin. Med. J. (Engl) **132**, 161–170 (2019). 10.1097/CM9.0000000000000054 30614867 10.1097/CM9.0000000000000054 PMC6365298

[81] T. Yi, Y. Song, L. Zuo, S. Wang, J. Miao, Front. Oncol. **11**, 646217 (2021). 10.3389/fonc.2021.646217 34249684 10.3389/fonc.2021.646217 PMC8267821

[82] Y. Xiong, S.C. Dowdy, A. Xue, J. Shujuan, N.L. Eberhardt, K.C. Podratz, S.W. Jiang, Gynecol. Oncol. **96**, 601–609 (2005). 10.1016/j.ygyno.2004.11.047 15721400 10.1016/j.ygyno.2004.11.047

[83] J. Zhang, J.Z. Huang, Y.Q. Zhang, X. Zhang, L.Y. Zhao, C.G. Li, Y.F. Zhou, H. Wei, J. Yu, EBioMedicine **53**, 102701 (2020) 10.1016/j.ebiom.2020.102701 10.1016/j.ebiom.2020.102701 PMC7063135 32151798

[84] S. Wang, W. Liu, Y. Ni, L. Wang, Y. Zhu, Q. Shi, Z. Yi, W. Wang, L. Liu, L. Yang, Y. Kuang, Y. Zhu, Q. Zhang, Z. Yang, J. Cancer **12**, 2550–2559 (2021). 10.7150/jca.54576 33854616 10.7150/jca.54576 PMC8040713

[85] J. Al-Maghrabi, A.S. Abdelrahman, T. Ghabrah, N.S. Butt, B. Al-Maghrabi, M.N. Khabaz, Pathol. Res. Pract. **213**, 348–352 (2017). 10.1016/j.prp.2017.01.012 28215640 10.1016/j.prp.2017.01.012

[86] I. Boutas, A. Kontogeorgi, C. Dimitrakakis, S.N. Kalantaridou, Mol. Biol. Rep. **48**, 5699–5705 (2021). 10.1007/s11033-021-06536-1 34241773 10.1007/s11033-021-06536-1

[87] C.J. Stewart, M.L. Crook, Int. J. Gynecol. Pathol. **29**, 555–561 (2010). 10.1097/PGP.0b013e3181e4ee4ea 20881856 10.1097/PGP.0b013e3181e4ee4ea

