# Supplementary material for: In-depth quantitative proteomics analysis revealed C1GALT1 depletion in ECC-1 cells mimics an aggressive endometrial cancer phenotype observed in cancer patients with low C1GALT1 expression
Source: Cell Oncol (Dordr). 2023 Feb 6;46(3):697–715. doi: 10.1007/s13402-023-00778-w (PMC10205863; doi:10.1007/s13402-023-00778-w)
Supplement: Supplementary file 5 — Supplementary Material 5 [file 13402_2023_778_MOESM5_ESM.pptx]

## Slide 1
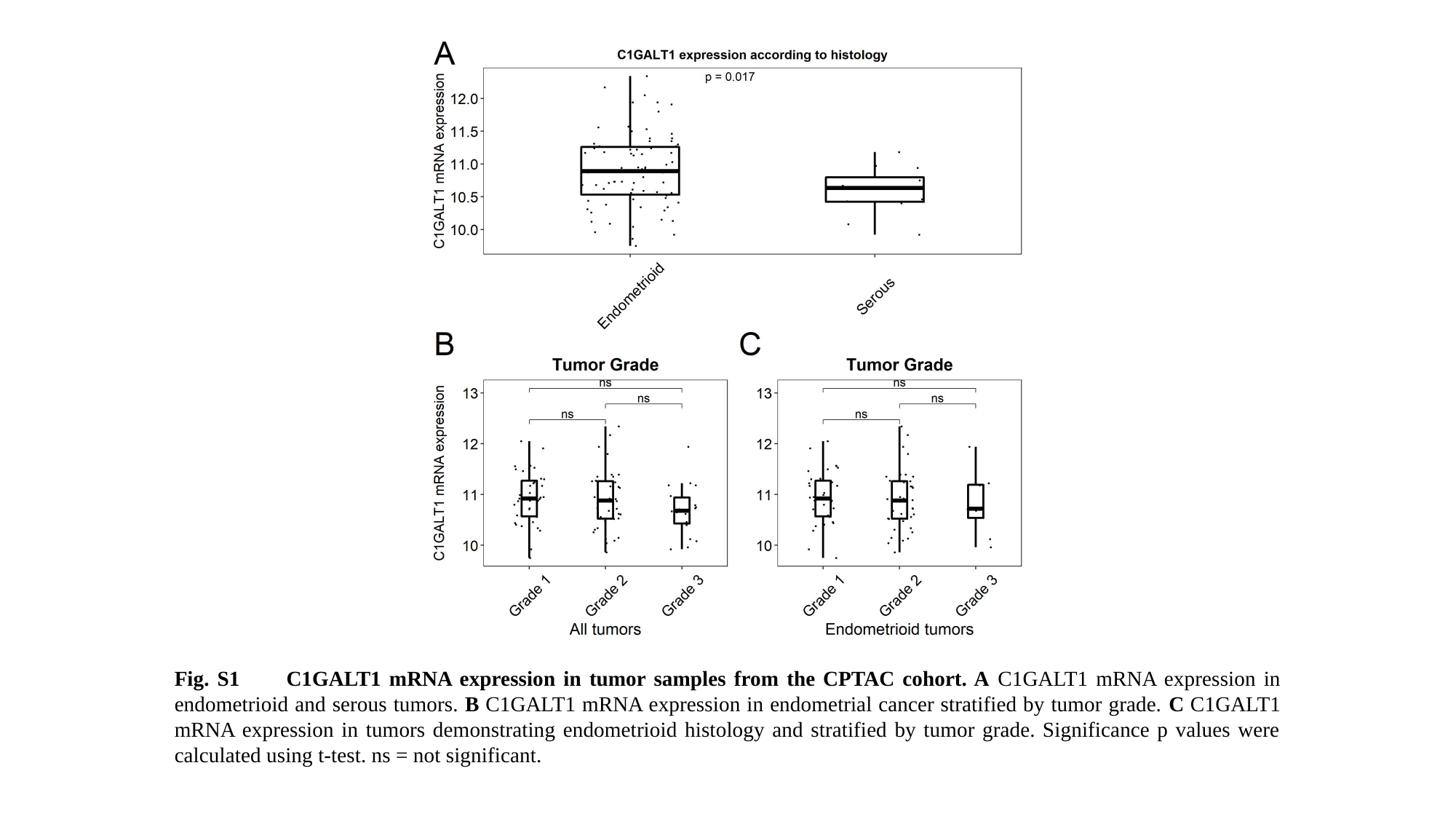

Fig. S1	C1GALT1 mRNA expression in tumor samples from the CPTAC cohort. A C1GALT1 mRNA expression in endometrioid and serous tumors. B C1GALT1 mRNA expression in endometrial cancer stratified by tumor grade. C C1GALT1 mRNA expression in tumors demonstrating endometrioid histology and stratified by tumor grade. Significance p values were calculated using t-test. ns = not significant.
