# Supplementary material for: In-depth quantitative proteomics analysis revealed C1GALT1 depletion in ECC-1 cells mimics an aggressive endometrial cancer phenotype observed in cancer patients with low C1GALT1 expression
Source: Cell Oncol (Dordr). 2023 Feb 6;46(3):697–715. doi: 10.1007/s13402-023-00778-w (PMC10205863; doi:10.1007/s13402-023-00778-w)
Supplement: Supplementary file 6 — Supplementary Material 6 [file 13402_2023_778_MOESM6_ESM.pptx]

## Slide 1
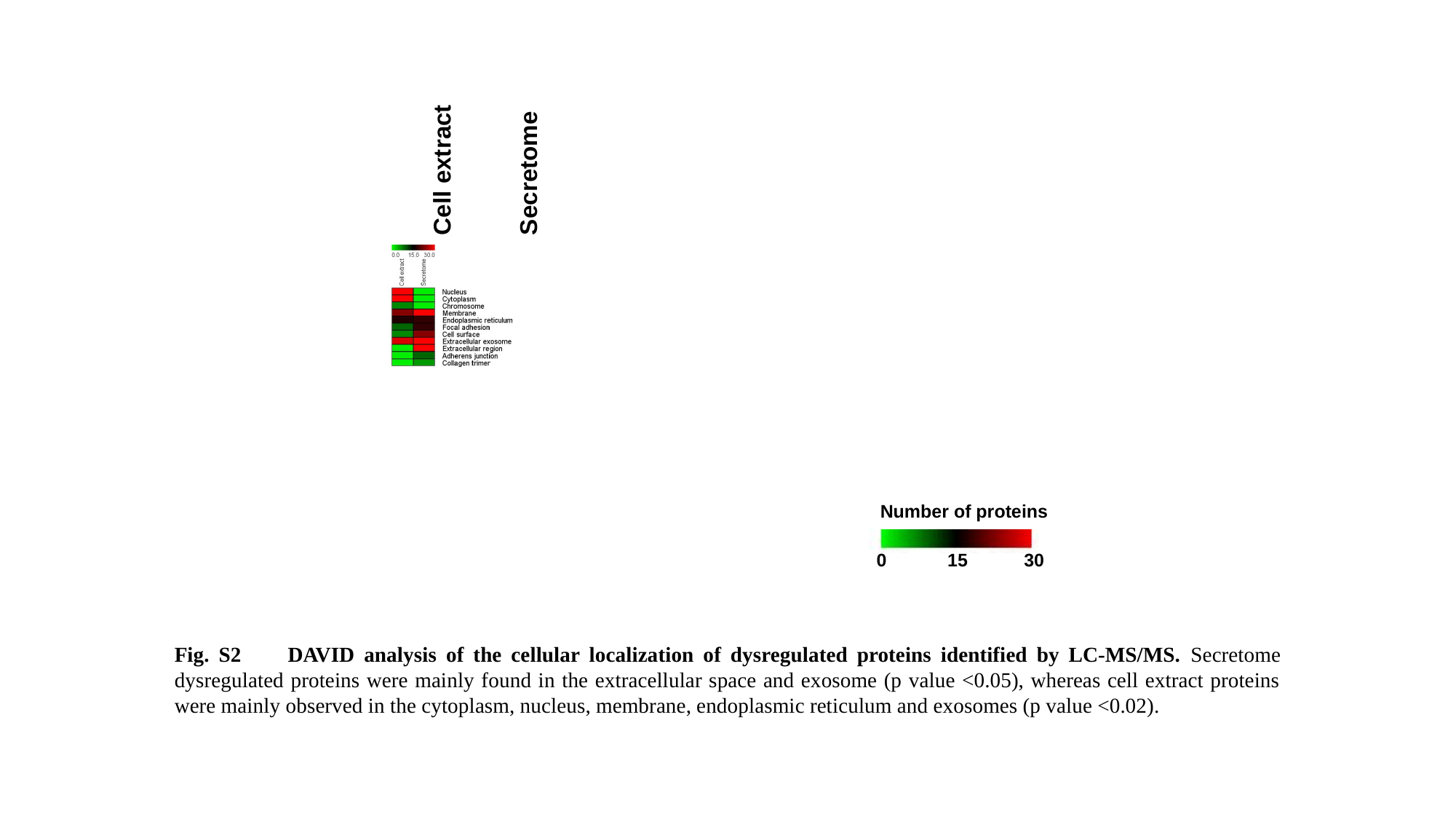

Cell extract
Secretome
Number of proteins
15
0
30
Fig. S2	DAVID analysis of the cellular localization of dysregulated proteins identified by LC-MS/MS. Secretome dysregulated proteins were mainly found in the extracellular space and exosome (p value <0.05), whereas cell extract proteins were mainly observed in the cytoplasm, nucleus, membrane, endoplasmic reticulum and exosomes (p value <0.02).
