# Supplementary material for: In-depth quantitative proteomics analysis revealed C1GALT1 depletion in ECC-1 cells mimics an aggressive endometrial cancer phenotype observed in cancer patients with low C1GALT1 expression
Source: Cell Oncol (Dordr). 2023 Feb 6;46(3):697–715. doi: 10.1007/s13402-023-00778-w (PMC10205863; doi:10.1007/s13402-023-00778-w)
Supplement: Supplementary file 7 — Supplementary Material 7 [file 13402_2023_778_MOESM7_ESM.pptx]

## Slide 1
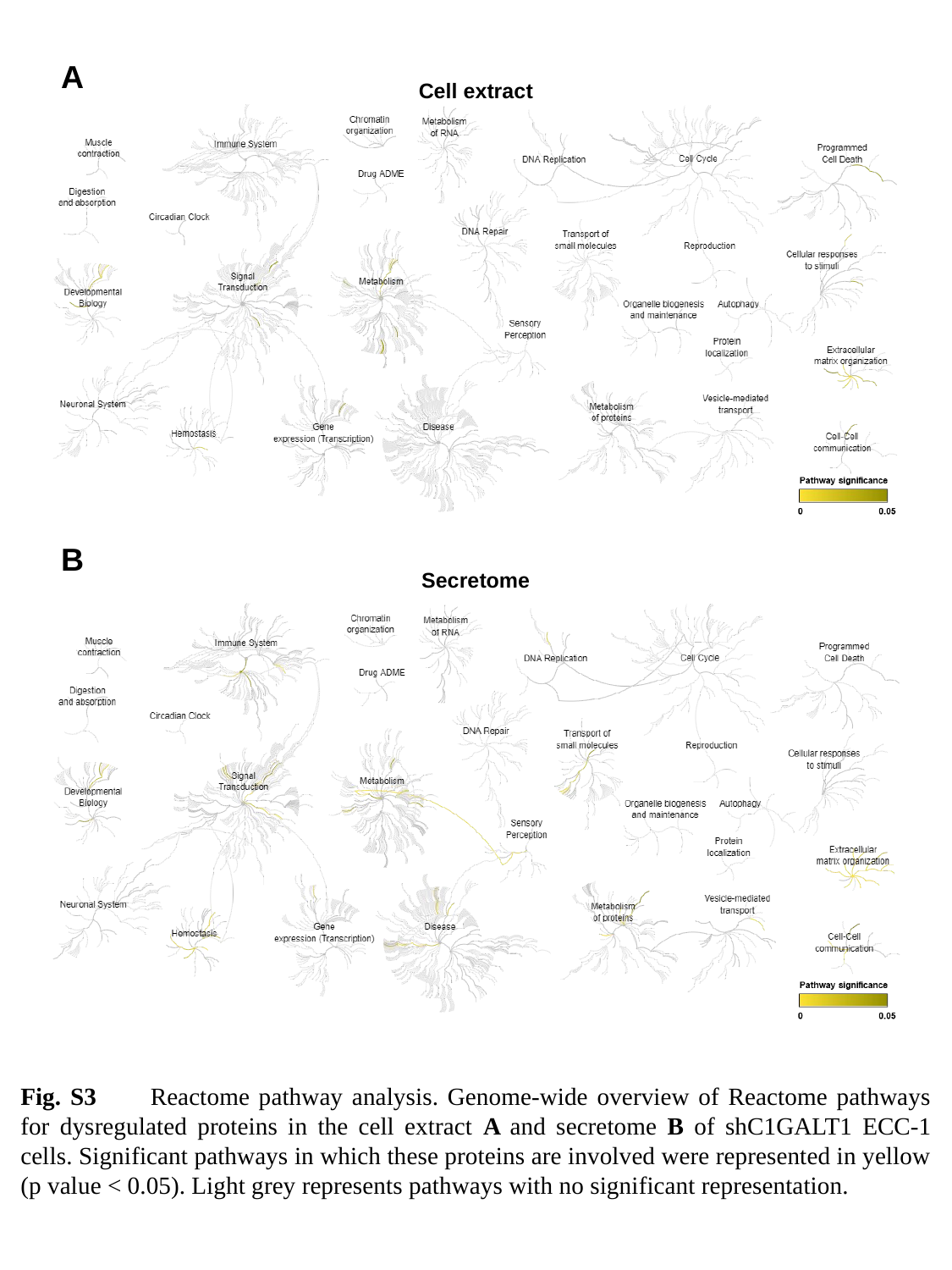

A
Cell extract
B
Secretome
Fig. S3	Reactome pathway analysis. Genome-wide overview of Reactome pathways for dysregulated proteins in the cell extract A and secretome B of shC1GALT1 ECC-1 cells. Significant pathways in which these proteins are involved were represented in yellow (p value < 0.05). Light grey represents pathways with no significant representation.
