# Supplementary material for: In-depth quantitative proteomics analysis revealed C1GALT1 depletion in ECC-1 cells mimics an aggressive endometrial cancer phenotype observed in cancer patients with low C1GALT1 expression
Source: Cell Oncol (Dordr). 2023 Feb 6;46(3):697–715. doi: 10.1007/s13402-023-00778-w (PMC10205863; doi:10.1007/s13402-023-00778-w)
Supplement: Supplementary file 8 — Supplementary Material 8 [file 13402_2023_778_MOESM8_ESM.pptx]

## Slide 1
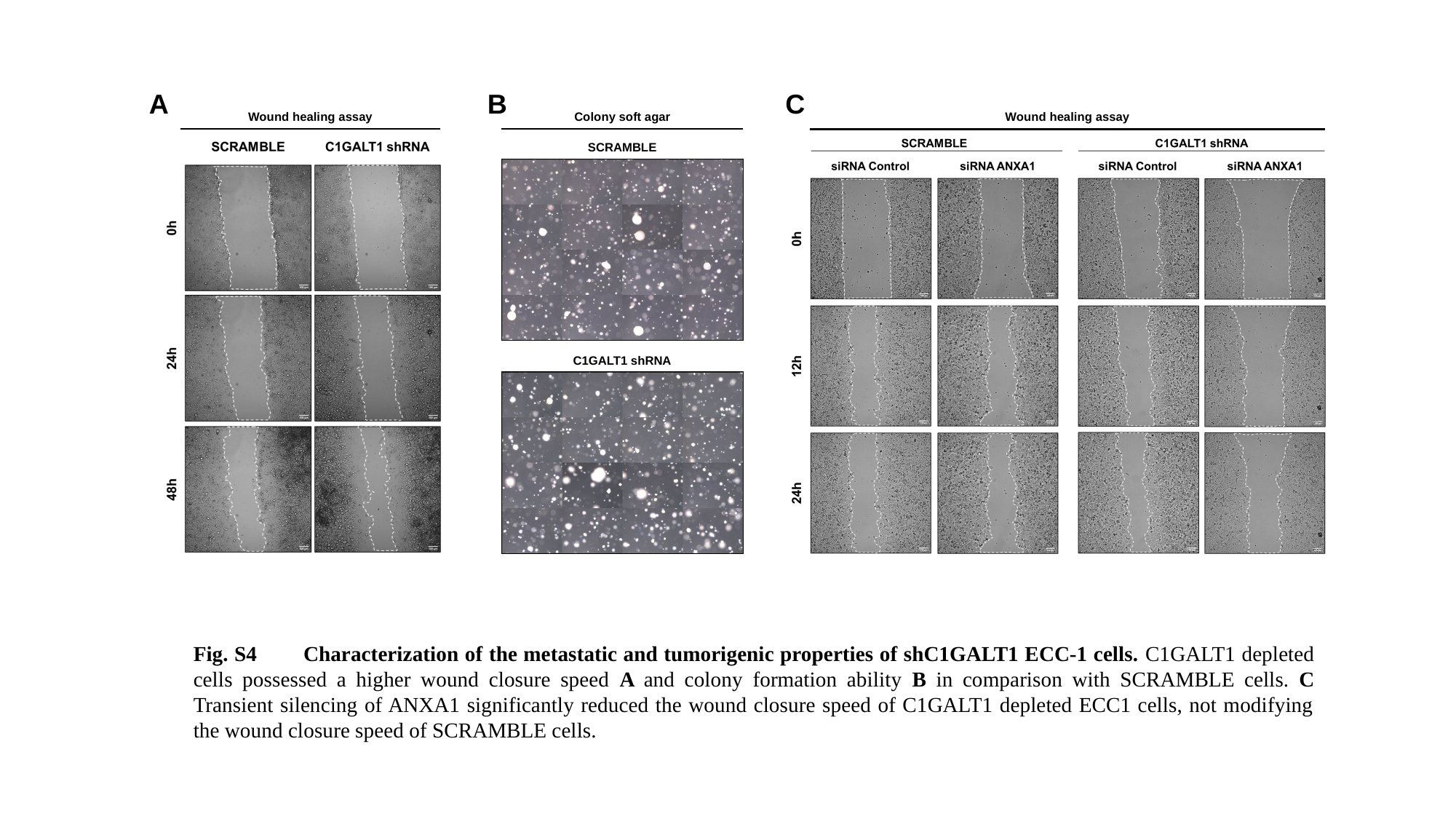

A
Wound healing assay
B
Colony soft agar
SCRAMBLE
C1GALT1 shRNA
C
Wound healing assay
Fig. S4	Characterization of the metastatic and tumorigenic properties of shC1GALT1 ECC-1 cells. C1GALT1 depleted cells possessed a higher wound closure speed A and colony formation ability B in comparison with SCRAMBLE cells. C Transient silencing of ANXA1 significantly reduced the wound closure speed of C1GALT1 depleted ECC1 cells, not modifying the wound closure speed of SCRAMBLE cells.
